# Revisiting the F3 Peptide: In Vitro Investigations of C- and N-Terminally Modified Peptide Conjugates for Radiotracer Development

**DOI:** 10.3390/ph19040558

**Published:** 2026-03-31

**Authors:** Maximilian Anderla, Marlene Grillmayr, Katharina Huemer, Thomas L. Mindt

**Affiliations:** 1 Institute of Inorganic Chemistry, Faculty of Chemistry, University of Vienna, Josef-Holaubek-Platz 2 and Währinger Straße 42, 1090 Vienna, Austria; 2 Vienna Doctoral School in Chemistry, University of Vienna, Währinger Straße 42, 1090 Vienna, Austria; 3 Ludwig Boltzmann Institute Applied Diagnostics, AKH Wien, Währinger Gürtel 18-20, 1090 Vienna, Austria; 4 Joint Applied Medicinal Radiochemistry Facility, University of Vienna and Medical University of Vienna, 1090 Vienna, Austria; 5 Department of Biomedical Imaging and Image Guided Therapy, Division of Nuclear Medicine, Medical University of Vienna, Währinger Gürtel 18-20, 1090 Vienna, Austria

**Keywords:** F3 peptide, nucleolin, indium-111, Auger electron therapy, optical/SPECT imaging

## Abstract

**Background/Objectives**: The F3 peptide, a tumor-homing peptide known to bind cell-surface nucleolin, is frequently employed as a targeting vector in cancer research. However, the impact of the modification site on its cellular binding properties has not been investigated yet. In this work, we aimed to design an improved F3-based radioconjugate by identifying the optimal conjugation site and establishing a protocol for its biological evaluation in vitro. To achieve this, we compared F3 peptide derivatives modified at their N- or C-termini with DOTA for complexation of indium-111 (^111^In) for SPECT or Auger electron therapy or a fluorophore (FITC) for optical imaging. **Methods**: N-and C-terminal DOTA-modified F3 peptides were radiolabeled with indium-111 and compared for their in vitro stability in different physiologically relevant media. Suitable nucleolin-positive cell lines for further in vitro studies were identified by confocal microscopy of a FITC-labeled F3 peptide derivative. The radioconjugates were then investigated on MDA-MB-231 (breast cancer) and PC-3 (prostate cancer) cells for nucleolin-specific cell binding and uptake, and several parameters of the in vitro assays were varied to establish a suitable protocol. **Results**: In general, in vitro assays with F3 peptide conjugates are challenging, as the outcome depends on a number of experimental parameters, leading, in some cases, to varying results. In particular, the presence of Ca^2+^ and Mg^2+^ had a decisive impact on the results, likely because the metal ions compete with the binding of F3 conjugates to nucleolin. The C-terminal modified, ^111^In-labeled F3 radioconjugate performed better than the N-terminal modified analog. While several parameters of the in vitro experiments were optimized, the overall cell uptake in vitro of radioactivity was still low (<2% of applied radioactivity). **Conclusions**: A standardized in vitro protocol for evaluating F3 peptide conjugates on cancer cells was established, revealing that the C-terminus is the preferred site for modification. Because the cellular uptake of the radiotracer was shown to likely not be sufficient for radiotracer development, further studies on the optimization of the F3 peptide conjugates, including structural modifications, are required.

## 1. Introduction

Targeted radionuclide therapy (TRT) is a rapidly expanding field in nuclear oncology, offering new applications in personalized precision medicine for the management of cancer [1]. TRT with Auger electron (AE) emitters holds particular promise due to the high linear energy transfer (LET) of AEs and their short range in biological matter [2]. This results in energy deposition within nanometer-scale distance of the decay event and therefore enables highly localized treatment with minimal collateral damage to surrounding, non-targeted healthy tissue [3].

DNA is considered the primary biological target for AE-induced cell death by triggering apoptosis through the induction of DNA double-strand breaks [4]. Thus, nuclear localization of the radionuclide is generally regarded as beneficial for achieving efficient therapeutic effects [5]. However, the subcellular range of AE requires elaborated delivery systems for enabling selective tumor targeting, cellular uptake, and intracellular transport to DNA within the cell nucleus [4,6].

Nucleolin (NCL) is a ubiquitous, multifunctional phosphoprotein that plays critical roles in cellular processes such as ribosome biogenesis, cell proliferation, and angiogenesis [7]. In healthy cells, NCL is predominantly localized in nucleoli within the nucleus, whereas in cancer cells, it is aberrantly expressed at the cell surface [7,8]. Consequently, NCL has received considerable attention as a biomarker and therapeutic target in cancer treatment [9,10,11,12,13,14]. NCL can act as a shuttle protein between the cell surface and the nucleus for suitable ligands, enabling efficient transport from the plasma membrane to the nucleus after endocytotic internalization [15,16].

Porkka et al. identified the tumor-homing F3 peptide (KDEPQRRSARLSAKPAPPKPEPKPKKAPAKK) in 2002 by screening a phage-displayed cDNA library [17]. Fluorescein-labeled F3 exhibited prominent nuclear accumulation in HL-60 (myeloid leukemia) and MDA-MB-435 (breast cancer) cells, a process shown to be mediated by cell-surface-expressed NCL [18]. This peptide has since been investigated as a tumor-targeting vector across diagnostic and therapeutic platforms, including conjugation to protein toxins [19,20,21,22], incorporation into nanoparticle delivery systems [23,24,25,26,27,28,29,30,31], and radiolabeling for diagnosis by nuclear imaging and TRT [32,33,34,35,36]. To date, the F3 peptide continues to be a vivid area of research in the field of radiotracer development (Figure S1).

Despite the growing body of publications, no systematic study has examined the optimal site of the F3 peptide for conjugating drugs or radioisotopes that preserves receptor-binding affinity and nucleus shuttling functionality. In addition, study outcomes of radiolabeled F3-based derivatives are considerably heterogeneous in terms of site of modification, radionuclides, and the cell and animal models used. For example, Essler et al. reported functionalizing a 32-amino acid F3 peptide at an N-terminal tyrosine with either DOTA (1,4,7,10-tetraazacyclododecane-1,4,7,10-tetraacetic acid) for actinium-225 (^225^Ac) or DTPA (diethylenetriaminepentaacetic acid) for bismuth-213 (^213^Bi) [37]. In a follow-up study by the same group, approximately 10% of the [^213^Bi]Bi-DTPA-F3 derivative bound to OVCAR-3 cells in vitro [33]. Conversely, Cornelissen et al. [35] observed around 2% binding in vitro to H2N cells for an ^111^In-labeled F3 derivative modified nonspecifically with DTPA at available amines (N-terminus and/or lysine residues).

Another study reports site-specific modification of the F3 peptide with NOTA (1,4,7-triazacyclononane-1,4,7-triacetic acid) at its ^5^Gln residue using an enzymatic approach [36]. Interestingly, this residue has previously been identified as important for tumor cell binding of a high-mobility group nucleosomal binding domain 2 (HMGN2) phage from which the F3 peptide is derived [17]. The resulting conjugate was radiolabeled with fluorine-18 (^18^F) using the aluminum [^18^F]fluoride–NOTA method ([^18^F]AlF) and displayed a reported binding affinity to H2N cells of approx. 46 nM. An iodine-125 labeled C-terminal modified F3 derivative was described by Bhojani et al., and evaluated in vitro (no absolute values for cellular uptake reported) and in vivo using MDA-MB-435 cells [32]. Drecoll et al. presented a dimeric construct consisting of N-terminally modified F3 peptides and DTPA [34]. After radiolabeling with ^213^Bi, this compound achieved relatively high tumor uptake in vivo (around 32% injected dose per gram; ID/g) following intraperitoneal (i.p.) administration into mice bearing i.p. MDA-MB-435 xenografts [34]. This value differs from the in vivo tumor uptake reported by Bhojani et al. and Cornelissen et al., who observed 1.05% ID/g and 0.8% ID/g, respectively [32,35]. The discrepancies may be attributable to the dimeric design of the ^213^Bi-conjugate used by Drecoll et al. [34] versus monomeric F3 conjugates used by the other groups, differences in the combination of chelator and radionuclide used, and differences in the administration route of the radiotracer (intraperitoneal versus intravenous), or a combination thereof.

**Figure 1:**
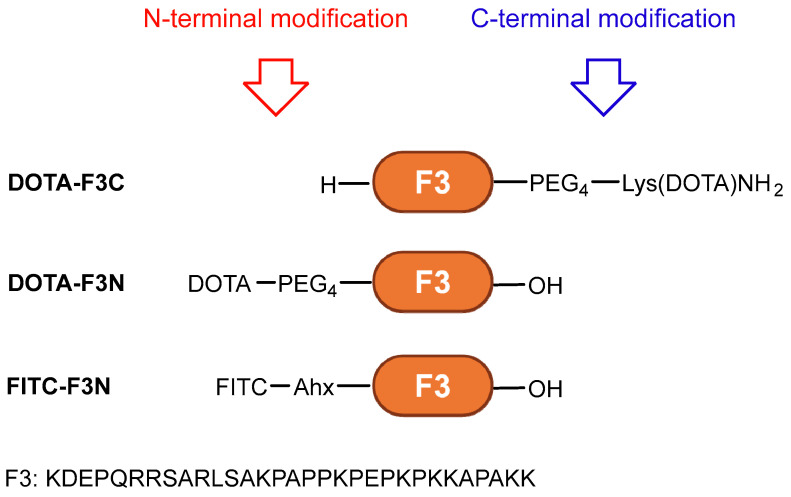
F3 peptide-based conjugates investigated in this work; PEG = polyethylene glycol, DOTA = 1,4,7,10-tetraazacyclododecane-1,4,7,10-tetraacetic acid, Ahx = aminohexanoic acid, and FITC = fluorescein isothiocyanate.

The F3 peptide represents an attractive ligand for AE radioendotherapy; however, further optimization is needed for clinical translation. In this study, we present a direct side-by-side comparison of site-specifically modified F3-based conjugates at either the N- or C-terminus (Figure 1). A fluorescently labeled analog was applied to pre-screen cell lines for uptake and subcellular localization using confocal microscopy. Derivatives functionalized with DOTA were radiolabeled with indium-111 (^111^In, *t*_1/2_ = 2.80 d, 100% EC, *E*_γ_ = 245 keV (94%), 171 keV (91%), 7.4 AE/decay) [5,38], employed here as a model radionuclide for AE emitters and SPECT (single-photon emission computed tomography) imaging. Their serum stability, as well as cellular binding and internalization, were evaluated in different cancer cell lines, with particular emphasis on in vitro assays designed to interrogate the F3-NCL interaction. The aim of this work was to, firstly, identify the best-suited conjugation site for modification of F3 and subsequently develop and investigate an F3-based radioconjugate for applications in nuclear medicine.

## 2. Results and Discussion

### 2.1. Conjugates

The full structures of all conjugates are provided in the Supporting Information (Figure S2). F3-PEG_4_-Lys(DOTA)NH_2_ (**DOTA-F3C**), DOTA-PEG_4_-F3 (**DOTA-F3N**), and FITC-Ahx-F3 (**FITC-F3N**) were sourced from commercial suppliers. A polyethylene glycol (PEG) linker was selected due to its hydrophilicity, flexibility, chemical inertness, metabolic stability, and minimal risk of toxicity or immunogenicity. The lysine on the C-terminus of the PEG linker enables convenient preparation of **DOTA-F3C** via solid-phase peptide synthesis. **FITC-F3N**, featuring an N-terminal fluorescein isothiocyanate (FITC) coupled to an aminohexanoic acid (Ahx) spacer, corresponds to the F3-derivative employed in the original report [17]. Across all conjugates, the spacers were incorporated to provide spatial separation between the imaging probe and the targeting vector to minimize potential interference with the binding of the conjugates to NCL.

### 2.2. Radiolabeling with [^111^In]InCl_3_

Radiolabeling of precursors **DOTA-F3C** and **DOTA-F3N** with [^111^In]InCl_3_ was performed in 0.3 M sodium acetate buffer (pH 4.5) at 95 °C for 12 min (Scheme S1). Radio-HPLC confirmed radiochemical yields and purities (RCYs and RCPs) of ≥95% (Figures S3 and S4). The resulting radioconjugates [^111^In]In-**DOTA-F3C** and [^111^In]In-**DOTA-F3N** were obtained at apparent molar activities (*A*_m_) of 2.8–56.6 MBq/nmol and were used for in vitro studies without further purification.

### 2.3. In Vitro Stability Studies and Serum Protein Binding

**Figure 2:**
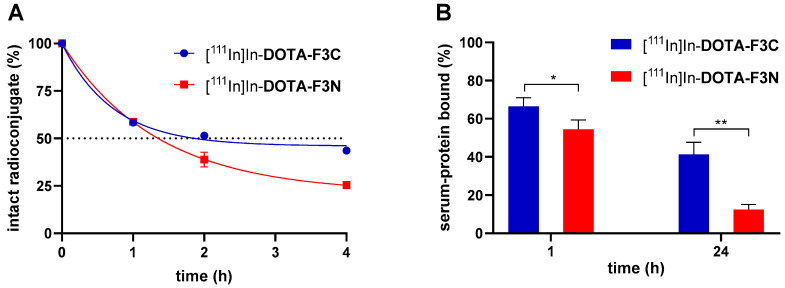
(**A**) Stability of ^111^In-labeled conjugates **DOTA-F3C** and **DOTA-F3N** in human serum at 37 °C. Data were normalized to 100% at t = 0 h (initial radiochemical purity ≥95%) and are presented as mean ± SD (n = 2). (**B**) Serum-protein bound fractions of ^111^In-labeled conjugates **DOTA-F3C** and **DOTA-F3N** at indicated time points. Data are presented as mean ± SD, * for *p* < 0.05, and ** for *p* < 0.01 (n = 3).

The ^111^In-labeled compounds **DOTA-F3C** and **DOTA-F3N** were incubated in human serum to determine their stability. Peptides composed of natural amino acids are generally susceptible to rapid enzymatic breakdown due to the high abundance of proteases in vivo [39]. While serum contains soluble proteases, it lacks membrane-bound enzymes found in epithelial cells of blood vessels and major organs such as the liver and kidneys [40,41]. Thus, in vitro serum stability assays provide only an approximation of a peptide’s in vivo stability. Samples were collected at predefined time points and analyzed by radio-HPLC (Figure 2A). Serum half-lives (*t*_1/2_) were determined by nonlinear regression (one-phase decay, Table 1) using GraphPad Prism 8.

The radioconjugate [^111^In]In-**DOTA-F3C** exhibited a prolonged *t*_1/2_ (2.4 h) compared to [^111^In]In-**DOTA-F3N** (1.5 h), indicating a stabilizing effect of *C*-terminal modification on peptide integrity in serum. Increased metabolic stability of a radioconjugate is typically correlated with enhanced tumor uptake and retention, making this property particularly advantageous for peptide-based vectors [42]. Both compounds produced a major metabolite at a similar retention time (*t*_R_~6 min), indicating formation of a common, not further identified radiolabeled fragment (Figures S5 and S6). Control experiments in phosphate-buffered saline (PBS) and cell medium (RPMI 1640) at 37 °C confirmed that all tested radioconjugates were sufficiently stable for at least 24 h (>90% intact), ensuring their suitability for subsequent in vitro cell assays (Table 1).

In parallel with the serum stability experiments, the interaction of the radiolabeled compounds with serum proteins was investigated. The serum protein–bound fractions of the ^111^In-labeled peptides **DOTA-F3C** and **DOTA-F3N** reached about 60% after 1 h (Figure 2B). Albumin, the most abundant serum protein, typically accounts for the majority of drug–protein interactions in blood [43]. The substantial binding of the radioconjugates to serum proteins observed likely reflects electrostatic interactions between the positively charged side chains of the peptide and negatively charged serum albumin at physiological pH [44]. For **DOTA-F3N**, the significantly lower association to serum proteins after 24 h compared with **DOTA-F3C** (12.5 ± 2.1% and 41.3 ± 5.1%, respectively) correlates with its faster degradation and subsequent loss of protein affinity of its metabolites.

### 2.4. Screening of Cancer Cell Lines

**Figure 3:**
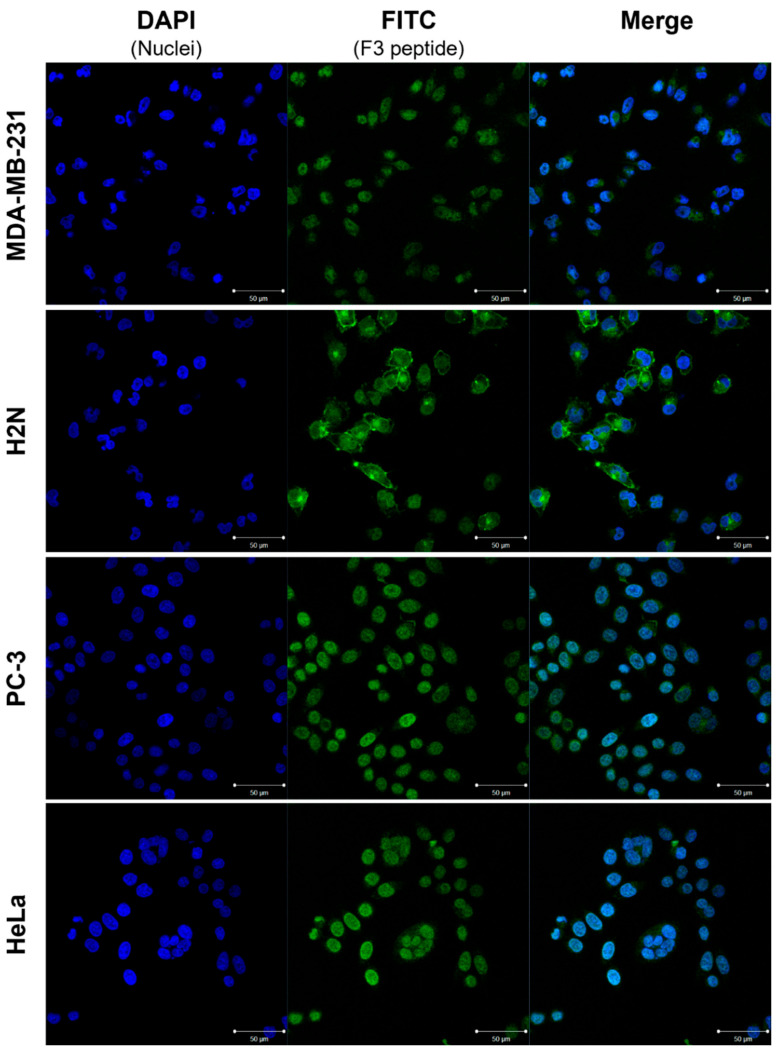
Confocal fluorescence images of indicated cell lines incubated with 10 nM **FITC-F3N** (green) for 60 min at 37 °C. Nuclei were counterstained with DAPI (blue). Scale bars: 50 µm.

To investigate the influence of the modification site of F3 peptide-based conjugates on cell binding and internalization properties, a suitable in vitro model is required. Accordingly, a panel of cancer cell lines was screened for uptake and subcellular distribution of the fluorescently labeled peptide **FITC-F3N**. The triple-negative breast cancer cell (TNBC) line MDA-MB-231 was selected due to its widespread use in the literature for NCL-targeting ligands [10,11,21,26,45,46,47]. Based on their recent use with radiolabeled F3 peptide derivatives, MDA-MB-231-H2N (H2N) cells were also included in the panel [35,36]. This breast cancer cell line is stably transfected with the human epidermal growth factor receptor 2 (*HER2*) gene. PC-3 (prostate cancer, bone metastasis), and HeLa (cervical cancer) cells were added due to their common application in related studies [8,19,20,48,49,50]. The MDA-MB-435 cell line, which was used in the first reports about the F3 peptide [17,18] was excluded due to its reported uncertain origin [51]. The selected cell lines were then incubated with **FITC-F3N**, and fluorescence localization was examined by confocal microscopy (Figure 3).

Confocal imaging confirmed nuclear allocation of fluorescence across all tested cell lines, consistent with NCL-mediated nuclear translocation after binding of the probe to NCL at the cell’s plasma membrane. While PC-3 and HeLa exhibited predominantly nucleus-localized signals, MDA-MB-231 cells showed more pronounced cytoplasmic accumulation. H2N cells displayed distinct membrane-associated fluorescence, in line with reported findings [35]. Despite intercellular variability in signal intensity and localization, each cell line demonstrated binding and internalization of the peptide conjugate **FITC-F3N**.

**Figure 4:**
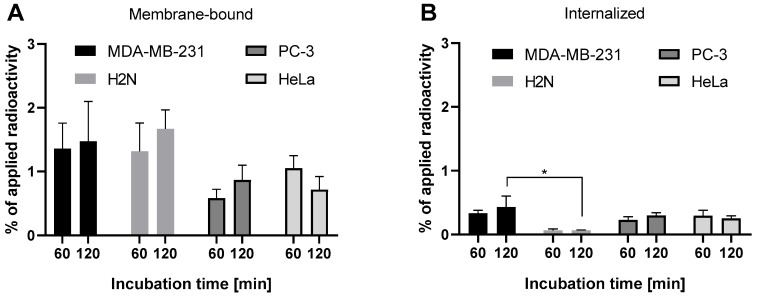
Results of cell uptake assay using different cancer cell lines (5 × 10^4^ cells/well for MDA-MB-231 and H2N cells, 6 × 10^4^ cells/well for PC-3 and HeLa cells, all in 24-well plates) incubated with [^111^In]In-**DOTA-F3C** (1 nM). (**A**) Membrane-bound fraction and (**B**) internalized fraction. Columns represent mean ± SD; * for *p* < 0.05 (n = 2–3, each in triplicate).

To assess whether the confocal imaging findings with **FITC-F3N** correlate with the uptake of F3-based radioconjugates, initial cellular uptake studies were conducted with ^111^In-labeled **DOTA-F3C**, as the C-terminus is the predominant site of modification of F3 derivatives reported in the literature [31,32,46]. Unlike optical methods, this assay enables quantification of unbound, membrane-bound, and internalized fractions through sequential washing with phosphate-buffered saline (PBS), treatment with an acidic glycine buffer, and final cell lysis with a sodium hydroxide solution. Serum-free RPMI 1640 lacking albumin, a widespread standard cell medium, was used for assays to avoid potential interactions with serum proteins as observed before (Figure 2B). MDA-MB-231, H2N, PC-3, and HeLa cells were screened for binding and internalization of [^111^In]In-**DOTA-F3C** (Figure 4).

Overall, cell-associated radioactivity was modest across all cell lines tested (total < 2%). MDA-MB-231 and H2N cells exhibited the highest membrane-associated radioactivity (~1.5%), whereas PC-3 and HeLa cells showed lower levels (~1%). H2N cells displayed the lowest internalized fraction (~0.1%), consistent with predominant membrane-binding observed in fluorescence imaging with **FITC-F3N** (Figure 3). This limited internalization poses a challenge for AE therapy, which requires nuclear delivery for effective DNA targeting [4,5,52,53]. Although Western blot analysis revealed lower total NCL levels in MDA-MB-231 cells compared to H2N, PC-3, and HeLa (Figure S7), elevated NCL levels did not necessarily correlate with enhanced binding or uptake of [^111^In]In-**DOTA-F3C**.

Of note, a direct comparison between fluorescence microscopy and radio-based cell uptake studies should be performed with caution because of the differences in structure between the compounds and the distinct features of each technique. The chemical nature of attached moieties, such as hydrophilic chelates or lipophilic fluorophores, can markedly influence the physicochemical properties and biological characteristics of the bioconjugates. While fluorescence imaging offers high spatial resolution and sensitivity, its quantitative accuracy is affected by factors such as photobleaching and quenching effects. In contrast, radio-based assays allow straightforward quantification of ligand uptake, but lack subcellular resolution [54,55,56]. For this reason, cell uptake in the present study could only be quantified using radiolabeled conjugates.

### 2.5. Experimental Variability and Reproducibility Challenges in Cellular Uptake Assays

Based on initial findings, MDA-MB-231 and H2N cells were selected for further evaluation of the ^111^In-labeled F3 peptide-based conjugates. Both cell lines exhibited similar levels of membrane binding but differed statistically significantly in their ligand internalization behavior, making them of particular interest for future studies on AE-induced therapeutic effects. However, uptake values frequently varied between individual experiments (see Section S6.1, Tables S1 and S2). Despite adjusting different experimental parameters, including cell numbers, apparent molar activity, and concentration of radioconjugates, no reliably reproducible improvement in cell uptake could be achieved. Blocking experiments in the presence of excess F3 to demonstrate NCL-specific binding were not effective. Overall, a tendency favoring C-terminal over N-terminal modification could be observed (Tables S1 and S2, entries 1, 2, 11, 12), but the overall issue with low radioactive uptake and reproducibility hindered conclusive conclusions.

These varying results highlight the challenges of in vitro analysis with radiolabeled F3 peptide conjugates and mirror the heterogeneous findings reported across the literature. Indeed, it has been noted that only modest levels of cell surface nucleolin were detected in vitro, while ligand binding was observed in vivo nevertheless [18]. This discrepancy warrants further investigation to establish a robust in vitro protocol. To better understand the F3 peptide–NCL interaction, subsequent studies focused on conjugate **DOTA-F3C**, based on the observed favorable trend, and on MDA-MB-231 cells. This cell line was prioritized over H2N cells for three main reasons:

(1) MDA-MB-231 cells have been recognized as a good model for cell-surface nucleolin expression by various research groups, whereas H2N cells have only been described in a limited number of studies; (2) internalization of tested conjugates was low in H2N cells, a key aspect to be examined in this project with regard to potential applications in AE therapy; (3) MDA-MB-231 cells represent triple-negative breast cancer (TNBC), a clinically relevant and treatment-resistant cancer cell subtype characterized by the lack of estrogen, progesterone, and *HER2* receptors. In contrast, *HER2*-transfected H2N cells no longer qualify as TNBC, further supporting the selection of MDA-MB-231 as the preferred cell model.

### 2.6. Effect of Calcium and Magnesium on Cell Binding and Internalization of F3-Based Radioconjugate

**Figure 5:**
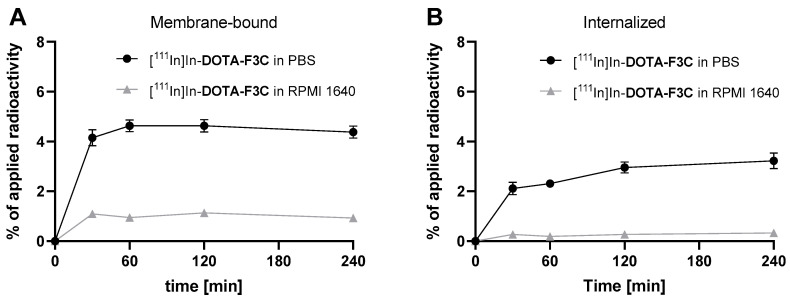
Cell uptake of [^111^In]In-**DOTA-F3C** (5 nM) in MDA-MB-231 cells (1 × 10^5^ cells/well in 6-well plates) conducted in different assay media. The medium was replaced either with PBS or RPMI 1640 medium (without FBS) 30 min prior to the addition of the radioconjugate, and incubation continued at 37 °C. (**A**) Membrane-bound fractions. (**B**) Internalized fractions. Data points show mean ± SD; assays were performed in triplicate.

In the course of preliminary experiments of this study, we observed that the cellular uptake of [^111^In]In-**DOTA-F3C** was considerably increased when the assay was performed in PBS instead of cell culture medium (see Section S6.2, Figure S8). To investigate these findings in more detail, additional experiments were performed with MDA-MB-231 cells. Cells were seeded in RPMI 1640 medium supplemented with 10% fetal bovine serum (FBS) and incubated with [^111^In]In-**DOTA-F3C** in either PBS or RPMI 1640 (without FBS) during the uptake assay (Figure 5).

Consistent with initial observations, cell uptake of [^111^In]In-**DOTA-F3C** was substantially higher under PBS conditions compared to RPMI 1640. The membrane-bound activity peaked at approximately 4.6% after 60 min, while internalization increased progressively, reaching about 3.3% after 4 h. For comparison, total cell uptake (membrane-bound plus internalized activity) under RPMI medium-based conditions remained low, reaching only approx 1.5% after 30 min.

In contrast to experiments with RPMI, it was observed that MDA-MB-231 cells began to detach from the culture plates upon incubation with PBS. As PBS lacks Ca^2+^ and Mg^2+^, the cells lost their characteristic spindle-shaped morphology and formed loosely attached clusters (Figure S9). These divalent metal cations are reported to be critical for the organization and clustering of integrins at adhesion sites, which are essential for stable cell attachment to the well-plate [57,58].

**Figure 6:**
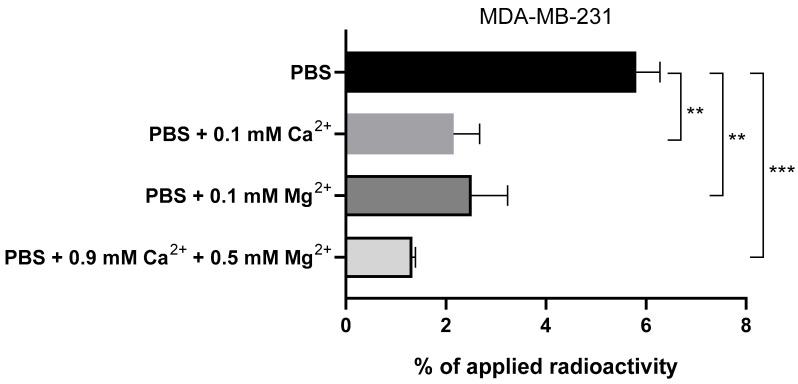
Cell-uptake of [^111^In]In-**DOTA-F3C** (5 nM) after 60 min in MDA-MB-231 cells (1 × 10^5^ cells/well in 6-well plates) seeded in RPMI 1640 medium supplemented with 10% FBS. The medium was replaced 30 min prior to the addition of the radioconjugate, using either PBS without supplements or supplemented with 0.1 mM Ca^2+^, 0.1 mM Mg^2+^, or 0.9 mM Ca^2+^ with 0.5 mM Mg^2+^, and incubation continued at 37 °C. Columns represent mean ± SD, ** for *p* < 0.01, and *** for *p* < 0.001 (multiple *t*-test; n = 2–3 in duplicate).

To assess the effect of Ca^2+^ and Mg^2+^ on radioligand uptake, a comparative assay was performed with [^111^In]In-**DOTA-F3C** in PBS with these ions at different concentrations and without them (Figure 6). Despite partial cell detachment, approximately 6% of the applied radioactivity remained bound to the cells in the absence of both metal cations. When 0.1 mM Ca^2+^ or Mg^2+^ was added, the binding decreased to 2–3%. In PBS containing 0.9 mM Ca^2+^ and 0.5 mM Mg^2+^, matching the concentrations found in RPMI 1640, the cell-associated activity dropped further to around 1%. These results closely align with uptake values obtained in RPMI 1640 (Figure 4 and Figure 5) and suggest that Ca^2+^ and Mg^2+^ interfere with the binding of F3-based radioconjugates to NCL. For subsequent experiments, PBS supplemented with 0.3 mM Mg^2+^ was selected as the preferred assay medium, as we observed that at this concentration, cell morphology and adhesion could be maintained while avoiding Ca^2+^-mediated interference.

This observation matches previous reports on the Ca^2+^-binding properties of NCL, which have been attributed to its highly acidic N-terminal domain [59]. Since the F3 peptide is also considered to interact with the N-terminal domain [18], we hypothesize that Ca^2+^/Mg^2+^ and the F3 peptide compete for similar or overlapping binding sites within NCL. Such competition may account for the reduced cellular uptake of the F3 peptide-based radioconjugate [^111^In]In-**DOTA-F3C** in the presence of higher concentrations of divalent cations like Ca^2+^ and Mg^2+^. The half-maximum binding of Ca^2+^ to NCL was measured at 105 µM [59], and the competitive interaction with [^111^In]In-**DOTA-F3C** suggests that this radioconjugate exhibits an affinity for NCL probably in the same range. A low affinity of the F3 radioconjugates can become an issue under physiological conditions, where Ca^2+^ (2.5 mM) and Mg^2+^ (0.9 mM) concentrations are substantially higher than those used in our experiments.

### 2.7. Other Parameters Investigated

We further examined the influence of the cell medium on the cellular uptake behavior of [^111^In]In-**DOTA-F3C**. For this purpose, MDA-MB-231 cells were seeded in EndoGRO™-VEGF (VEGF: vascular endothelial growth factor) medium and subsequently assayed under different buffer conditions (Figure S10). EndoGRO™-VEGF medium is a commercially optimized formulation that promotes rapid proliferation and contains multiple growth factors and additives. When cells were grown in this medium, total cell-associated activity increased more than threefold compared with RPMI 1640, with up to ~11% membrane-bound and ~3% internalized activity in PBS containing only Mg^2+^, whereas the presence of Ca^2+^ and Mg^2+^ again reduced uptake, in line with earlier observations. These findings suggest that specific medium components can promote NCL translocation to the cell surface and thereby enhance binding of the F3-based radioconjugate, although VEGF alone does not appear sufficient to account for this effect (Figure S11).

Since NCL is actively transported to the cell surface of proliferating cells [8,18,60,61], we further examined the impact of cell density on [^111^In]In-**DOTA-F3C** uptake, using EndoGRO™-VEGF medium to ensure adequate signal (Figure S12). Lower seeding densities yielded higher normalized uptake (~8% total bound at 0.5 × 10^5^ cells versus ~5% at 4 × 10^5^ cells), and optimal confluency was set at 60%. Very low seeding densities were excluded from further experiments due to insufficient confluency (<50%).

Finally, we observed that prolonged cell culture could be associated with reduced uptake of [^111^In]In-**DOTA-F3C**, with total binding declining from ~5% at passage 41 to ~2% at passage 43 in one representative cell aliquot (Figure S13). Although no uniform relationship between passage number and uptake was evident across all conducted experiments, this variability could contribute to the varying in vitro data reported and also observed during our studies (Tables S1 and S2, entries 3, 4, 11, 12). We therefore recommend early passage numbers and testing each new cell aliquot in preliminary scouting assays prior to extensive in vitro and in vivo studies.

Despite encouraging results with EndoGRO™-VEGF, this medium was not included in further studies due to restricted availability and its relatively artificial and complex composition. It should be noted that the mentioned parameters in this chapter were observed trends and could not be fully established due to limited reproducibility.

### 2.8. Standardized In Vitro Protocol to Interrogate F3/NCL Ligand–Receptor Interaction

The protocol involved seeding cells at a low passage number (<15) with 60% confluency, corresponding to 2 × 10^4^ MDA-MB-231 or 3 × 10^4^ PC-3 cells per well (24-well plates) in RPMI 1640 supplemented with 10% FBS, 24 h prior to the assay. The medium was replaced with PBS + 0.3 mM Mg^2+^ 30 min before the addition of the radioconjugate. For blocking studies, a 30 min preincubation at 37 °C with a 5000-fold molar excess of F3 peptide proved effective.

These conditions enabled a more reliable in vitro evaluation of F3 peptide-based radioconjugates. However, as this protocol was established with MDA-MB-231 and PC-3 cells, it is recommended that, before usage of other cells lines, the influence of the mentioned parameter is explored.

### 2.9. Comparison of C-Terminal Versus N-Terminal Modified F3 Peptide-Based Radioconjugates

**Figure 7:**
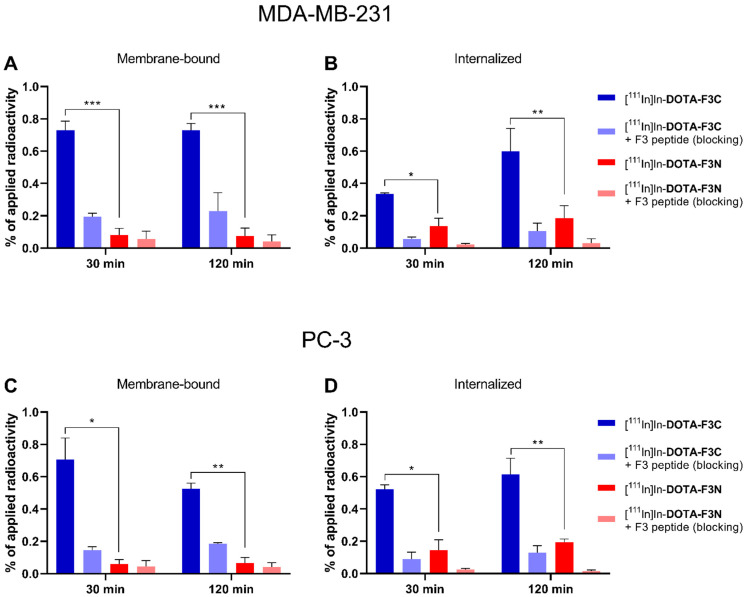
Comparison of cell-uptake of ^111^In-labeled conjugates **DOTA-F3C** and **DOTA-F3N** (5 nM/well, A_m_ = 4–13 MBq/nmol) in MDA-MB-231 cells (**A**,**B**) and PC-3 cells (**C**,**D**). The medium was replaced with PBS containing 0.3 mM Mg^2+^ 30 min prior to the addition of the radioconjugates; for blocking experiments, a 5000-fold excess of F3 peptide (25 µM) was included. Columns represent mean ± SD, * for *p* < 0.05, ** for *p* < 0.01, and *** for *p* < 0.001(multiple *t* test; n = 2–3, each in triplicate).

With the standardized in vitro protocol in hand, we compared side-by-side the cell-binding and internalization properties of the ^111^In-labeled conjugates **DOTA-F3C** and **DOTA-F3N**. To enable a direct comparison, both compounds were evaluated in parallel under identical conditions and with identical passage numbers of the same cell aliquots. MDA-MB-231 (breast cancer) and PC-3 (prostate cancer) cells were examined to assess NCL-specific binding and uptake of the radioconjugates in tumor cell lines of distinct origin (Figure 7).

Receptor specificity was confirmed by blocking experiments, in which cells were preincubated for 30 min with a 5000-fold excess of F3 peptide (25 µM). Notably, the C-terminally modified conjugate [^111^In]In-**DOTA-F3C** exhibited significantly increased cellular uptake in both tested cell lines compared with its N-terminally modified analog [^111^In]In-**DOTA-F3N**. After 30 min, the membrane-bound fraction of [^111^In]In-**DOTA-F3C** was 0.73 ± 0.04% in MDA-MB-231 and 0.71 ± 0.10% in PC-3 cells. This represents an approximately 10-fold increase over [^111^In]In-**DOTA-F3N** (≤0.08% membrane-binding in both cell lines). The internalized fraction of [^111^In]In-**DOTA-F3C** after 120 min reached 0.60 ± 0.10% and 0.62 ± 0.10% in MDA-MB-231 and PC-3 cells, respectively. Relative to [^111^In]In-**DOTA-F3N**, this corresponds to a threefold increase (0.19 ± 0.06% and 0.21 ± 0.01% in MDA-MB-231 and PC-3 cells, respectively).

These results demonstrate that C-terminal modification of the F3 peptide is preferred over N-terminal modification in terms of cell binding and uptake. With the superior binding properties of [^111^In]In-**DOTA-F3C** in mind, the distinct distribution profile between membrane-bound and internalized fractions compared with its N-terminally modified analog is noteworthy—approximately 60% of the total bound activity of [^111^In]In-**DOTA-F3C** was membrane-associated, whereas for [^111^In]In-**DOTA-F3N**, only around 20% remained membrane-associated, and 80% was internalized. This pattern indicates that the N-terminal amino acids of the F3 peptide are involved in NCL recognition, while the C-terminus may contribute to subsequent internalization processes.

Considering that the F3 peptide has been reported by several groups to target cell-surface NCL across various cancer cell lines, this ligand–receptor pair is of interest for targeted cancer therapy. Even though our study could optimize several parameters for in vitro investigations, absolute cellular uptake remained low for both tested radioconjugates, with total <2% of the applied radioactivity associated with cells (membrane-bound and internalized). For the development of an F3 peptide-based radiopharmaceutical, its NCL binding and cellular uptake properties would need to be improved.

## 3. Materials and Methods

### 3.1. General Information

High-performance liquid chromatography (HPLC) analyses were performed using Milli-Q water (18.2 MΩ·cm, Milli-Q Advantage System, Merck, Darmstadt, Germany). Unless otherwise stated, all chemicals and solvents were obtained from commercial suppliers and used without further purification.

Phosphate-buffered saline (PBS; Thermo Fisher Scientific, Waltham, MA, USA, cat. no. 14190144) was supplemented, where indicated, with CaCl_2_·2H_2_O and/or MgCl_2_·6H_2_O (both from Sigma-Aldrich, St. Louis, MO, USA, cat. no. C3306 and M0250, respectively). EndoGRO™-VEGF was from Merck (Darmstadt, Germany) (cat. no. SCME002). Recombinant human VEGF was from R&D Systems (Minneapolis, MN, USA) (cat. no. 293-VE-010/CF). Pooled human serum (“off the clot”) was purchased from Innovative Research (Novi, MI, USA).

**Radiolabeling** was performed using a thermoshaker (Eppendorf™ Thermomixer C, Eppendorf SE, Hamburg, Germany). [^111^In]InCl_3_ was obtained from Curium (Petten, The Netherlands) in medical-grade quality. Activities were registered by an iDose Standard Ionisation Chamber from Elysia Raytest or a VDC-405 dose calibrator from Comecer. In vitro experiments were carried out with radiolabeled compounds with a purity of at least 95% (radio-HPLC). Samples were counted for 30 s using a dynamic energy window on a 2480 Wizard2^®^ γ-counter from PerkinElmer (Waltham, MA, USA).

**Reversed phase radio-HPLC analysis** of radiolabeled conjugates was performed with a XSelect™ Premier Peptide CSH C18 (Waters, Milford, MA, USA; 130 Å, 2.5 μm, 2.1 × 150 mm) on an Agilent 1260 Infinity II (VW UV-Vis detector) (Agilent Technologies, Santa Clara, CA, USA) paired with a GABI Nova detector (Elysia Raytest, Straubenhardt, Germany). The flow rate was maintained at 0.3 mL/min, with UV detection at λ = 220 nm. The mobile phases were: A = Milli-Q water and B = acetonitrile, each with 0.1% TFA.

The following method was used (A + B = 100%, total run time = 15 min): 0–0.5 min, 5% B; 0.5–10 min, to 40% B; 10–10.5 min, to 95% B; 10.5–12.5 min, 95% B; 12.5–13 min, 5% B.

**Reversed-phase HPLC-MS** (LC-MS) analyses were conducted on an Agilent 1260 Infinity II system equipped with a Flexible pump, a 1260 VWD UV-Vis detector (λ = 220 nm), and the LC–MSD system using an Acquity UPLC^®^ BEH C18 column (300 Å, 1.7 μm, 2.1 mm × 100 mm) (Waters, Milford, MA, USA). Mobile phases of Milli-Q water (A) and acetonitrile (B), each containing 0.1% formic acid, were used as eluents. A constant flow rate of 0.6 mL/min and a column temperature of 20 °C were maintained throughout the analysis. The following gradient was used: 0–0.5 min, 5% B; 0.5–6.5 min, to 95% B; 6.5–7.5 min, 95% B; 7.5–7.6 min, to 5% B; 7.6–8.0 min, 5% B.

**Stability studies** were conducted on an Agilent 1260 Infinity II system with a diode array detector (DAD) with a Gamma detector HERM LB 500 from Berthold Technologies (Bad Wildbad, Germany), using a Synergi^®^ Fusion-RP column (Phenomex; 80 Å, 4 μm, 250 × 10 mm). The mobile phase consisted of Milli-Q-H_2_O (A) and acetonitrile (B), each containing 0.1% trifluoroacetic acid (TFA). The flow rate was maintained at 3 mL/min with the column temperature set to 20 °C, and the following gradient was used (A + B = 100%, total run time: 24 min): 0–2 min, 10% B; 2–12 min, to 60% B; 12–12.5 min, to 95% B; 12.5–17.5 min, 95% B; 17.5–18 min, to 10% B.

**Confocal microscopy** was performed using an LSM 800 from Zeiss (Oberkochen, Germany) with Zen 3.4 (blue edition) software. Excitation wavelengths were 405 nm for DAPI, and 495 nm for FITC.

### 3.2. F3-Based Conjugates

F3-PEG_4_-Lys(DOTA)NH_2_ (**DOTA-F3C**) and DOTA-PEG_4_-F3 (**DOTA-F3N**) were obtained from Macrocyclics (Plano, TX, USA), while the F3 peptide and FITC-Ahx-F3 (**FITC-F3N**) were purchased from Biomatik (Cambridge, ON, Canada). All conjugates were supplied at >95% purity as verified by reversed-phase HPLC and mass spectrometry (manufacturer’s certificate of analysis).

### 3.3. Confocal Microscopy

Cells (1 × 10^4^/well) were seeded in RPMI 1640 + 10% FBS onto 8-well chamber slides (ibidi, cat. 80826) 24 h prior to the experiment. Medium was replaced with 200 µL RPMI 1640 (without FBS) containing 10 nM **FITC-F3N**, and cells were incubated for 1 h at 37 °C. Following incubation, the medium was aspirated, and the cells were washed twice with PBS. Cells were then fixed in 4% paraformaldehyde/PBS (pH 7.4) for 15 min at room temperature. After PBS washes, nuclei were counterstained with DAPI (0.5 µg/mL in PBS, 10 min, room temperature), followed by an additional PBS wash. Slides were coverslipped with mounting medium (ibidi, cat. 50001) and imaged by confocal microscopy.

### 3.4. Determination of F3-Based Conjugate Stock Concentration

Concentrations of stock solutions containing either the F3 peptide, **DOTA-F3C** or **DOTA-F3N** in Milli-Q H_2_O were quantified using a microvolume UV/vis spectrophotometer (NanoDrop One^C^, Thermo Fisher Scientific). Measurements were performed in triplicate, after blanking with Milli-Q water. For each measurement, 2 µL sample was applied, and the absorbance was determined at λ = 205 nm. The extinction coefficient (*ε*_205_) for each conjugate was calculated by summing the sequence-based extinction coefficient, as previously reported [62], and adding 2780 M^−1^ cm^−1^ for each additional amide bond (e.g., for a conjugated DOTA residue). The sequence-based *ε*_205_ for the F3 peptide was calculated at 87,850 M^−1^ cm^−1^. **DOTA-F3C** contains four additional amide bonds, resulting in a calculated *ε*_205_(DOTA-F3C) = 98,970 M^−1^ cm^−1^, whereas **DOTA-F3N** contains 2 additional amide bonds, giving a calculated *ε*_205_(DOTA-F3N) = 93,410 M^−1^ cm^−1^. Final concentrations were determined according to the Beer–Lambert law:

$$c=\frac{A}{\varepsilon *l}$$

where *c* is the concentration (mol/L), *A* is absorbance, *ε* is the extinction coefficient (M^−1^ cm^−1^), and *l* is the path length (cm).

### 3.5. Radiolabeling of DOTA-F3C and DOTA-F3N with [^111^In]InCl_3_

All procedures were performed in protein-low-binding Eppendorf tubes (Eppendorf SE, Hamburg, Germany). Stock solutions (1 mM) were prepared by dissolving the respective conjugates in Milli-Q water. Additonally, 1–5 µL (1–5 nmol) of conjugate was added to 50 µL of [^111^In]InCl_3_ (0.05 M HCl, ~3–50 MBq), followed by 10 µL of freshly prepared 0.3 M sodium acetate solution to achieve a pH of ~4.5. The mixture was heated at 95 °C for 12 min in a thermoshaker and was agitated at 400 rpm. An aliquot was subsequently analyzed by radio-HPLC. Details on instrumentation and the methods are provided in the general information. Radiolabeled conjugates eluted between approximately 7.1 and 7.3 min, while free ^111^In appeared at around 1.8 min. The radioconjugates were obtained with radiochemical yields and purities exceeding 95% (Figures S3 and S4) and were used for in vitro experiments without further purification.

### 3.6. Stability Studies and Serum Protein Binding

To assess stability, 50 μL (approximately 8 MBq) of the ^111^In-labeled compound in PBS was added to 950 µL of prewarmed RPMI 1640 (0% FBS, 1% PenStrep), PBS, or pooled human serum to achieve a final compound concentration of 250 nM. Samples were incubated at 37 °C with agitation. At designated time points, 100 µL aliquots of the incubated sample were taken out for analysis. For pooled human serum, proteins were precipitated by adding 100 µL ACN, followed by vortexing and centrifugation to yield a pellet and a supernatant. The supernatant was filtered, diluted 1:1 (*v*/*v*) with H_2_O + 0.1% TFA, and analyzed by radio-HPLC. Details on instrumentation and the methods are provided in the general information. For all other media, the aliquot was directly diluted with 100 µL H_2_O + 0.1% TFA, filtered, and analyzed by radio-HPLC. The injection volume for all samples was 70 µL.

For serum protein binding analysis, an additional 100 μL aliquot of the serum mixture was used as a control for total activity. The pellet was washed twice with PBS, with centrifugation after each wash. Residual radioactivity in the pellet was quantified using a γ-counter, and serum protein binding was calculated as the percentage of pellet-associated activity relative to the total activity measured in the control.

### 3.7. Cell Culture and Cell Uptake Assays with Standardized Protocol

Cell culture was performed using the following cell lines: MDA-MB-231 and PC-3 cells, both obtained from the American Type Culture Collection. HeLa cells were kindly provided by Dr. Michael Jakupec (University of Vienna), MDA-MB-231-H2N (H2N) cells were a generous gift from Dr. Bart Cornelissen (University of Oxford). Cells were grown as monolayers and maintained under standardized conditions in a humidified incubator at 37 °C with an atmosphere of 95% air and 5% CO_2_. Cultures were passaged upon reaching 80–90% confluency. Mycoplasma contamination was routinely monitored using the MycoAlert™ Mycoplasma Detection Kit (Lonza, Basel, Switzerland) following the manufacturer’s instructions. HUVECs were purchased from Promocell (Heidelberg, Germany) and were cultured in EndoGRO™ medium supplemented with the VEGF kit, consisting of recombinant (rh) VEGF (5 ng/mL), rh endothelial growth factor (5 ng/mL), rh fibroblast growth factor basic (5 ng/mL), rh insulin-like growth factor-1 (15 ng/mL), ascorbic acid (50 µg/mL), hydrocortisone hemisuccinate (1 µg/mL), heparin sulfate (0.75 U/mL), *L*-glutamine (10 mM), and 2% fetal bovine serum (FBS). In addition, this medium was supplemented with 100 U/mL penicillin and 100 µg/mL streptomycin (1% PenStrep). All other cells were cultured in Roswell Park Memorial Institute (RPMI) 1640 medium, supplemented with 1% PenStrep and 10% fetal bovine serum (FBS), which was all purchased from Gibco™ (Thermo Fisher Scientific, Waltham, MA, USA).

For cell uptake assays, MDA-MB-231 cells (2 × 10^4^ cells/well) or PC-3 cells (3 × 10^4^ cells/well) were seeded in 24-well plates 24 h prior to the experiment in RPMI 1640 + 10% FBS. In addition, 30 min prior to the start of the assay, culture medium was replaced with PBS supplemented with 0.3 mM Mg^2+^ (0.48 mL/well) and incubation was continued at 37 °C. For blocking experiments, a 5000-fold molar excess (25 µM) of blocking agent (F3 peptide) was added. ^111^In-labeled conjugates were then added to the cells (2.5 pmol in 20 µL; final concentration/well: 5 nM) and incubated at 37 °C for the indicated time points. The total volume was always 0.5 mL/well. After the defined incubation time, the medium was removed, and the cells were washed twice with PBS (0.5 mL/well). The combined supernatants represented the unbound radioligand.

Membrane-bound radioligand was removed by incubating cells on ice with ice-cold acidic glycine buffer (100 mM NaCl, 50 mM glycine, pH 2.8; 2 × 1 mL, 5 min), and supernatants were combined for quantification of receptor-bound radioactivity. Cells were then lysed by incubation with 1 M NaOH (1 mL, 10 min, room temperature), followed by washing steps with 1 M NaOH (2 × 1 mL 1 M NaOH). The lysate and cell rinse were collected to quantify internalized radioligand.

Standards were prepared in triplicate by spiking the same amount of radioconjugate added to each well (2.5 pmol in 20 µL) into 0.48 mL PBS in an Eppendorf tube. The amount of radioactivity of each fraction was quantified in a γ-counter and calculated as a percentage of the applied dose. Data and statistical analysis were performed with GraphPad Prism 8.0™.

### 3.8. Protein Isolation and Western Blot

Whole-cell lysates were prepared from 80 to 90% confluent monolayers cultured in 175 cm^2^ flasks. Protein extraction was performed using radioimmunoprecipitation assay (RIPA) buffer (Thermo Fisher Scientific, cat. 89990) supplemented with protease inhibitor cocktail (1:10; Sigma Aldrich, cat. P2714), in accordance with the manufacturer’s instructions. Protein concentrations were quantified using the Pierce™ BCA Protein Assay Kit (Thermo Fisher Scientific, cat. 23225). Samples were diluted in 4× Laemmli buffer (Bio-Rad, Hercules, CA, USA, cat. 1610747) containing 10% 2-mercaptoethanol (Bio-Rad, cat. 1610710), heated at 95 °C for 5 min, and resolved by SDS-PAGE on Mini-PROTEAN^®^ TGX™ 4–15% precast gels (Bio-Rad, cat. 4561083) with 15 µg protein per lane, using Tris/glycine/SDS buffer (Bio-Rad, cat. 1610732).

Proteins were transferred to nitrocellulose membranes (Trans-Blot^®^ Turbo™ Transfer Kit, Bio-Rad, cat. 1704270) using a Trans-Blot^®^ Turbo™ Transfer System (25 V, 30 min; Bio-Rad). Membranes were blocked for 1 h at room temperature with 2% bovine serum albumin (BSA; Sigma Aldrich, cat. A9647) in Tris-buffered saline containing 0.1% Tween-20 (TBST; 49.6 mM Tris, 137.9 mM NaCl, 0.1% Tween-20; pH 7.4; Sigma Aldrich). Membranes were incubated at 4 °C overnight with primary rabbit monoclonal anti-NCL antibody (1:2000; clone D4C7O, Cell Signaling Technology, Danvers, MA, USA) and rabbit polyclonal anti-β-actin antibody (1:2500; clone ab8227, Abcam, Cambridge, UK) diluted in 2% BSA/TBST. Following TBST washes, membranes were incubated for 1 h at room temperature with goat anti-rabbit IgG-HRP secondary antibody (1:2500; Thermo Fisher Scientific, cat. A16104) in 2% BSA/TBST and washed again.

Protein bands were visualized using Pierce™ ECL Western Blotting Substrate (Thermo Fisher Scientific, cat. 32106) and imaged with a ChemiDoc System (Bio-Rad). Chemiluminescence signals were detected at 425 nm, and images were processed using Image Lab 6.0 software (Bio-Rad).

## 4. Conclusions

Our studies show that C-terminally modified [^111^In]In-**DOTA-F3C** outperforms the N-terminal analog in terms of serum half-life, cellular binding, and uptake, also showing that MDA-MB-231 and PC-3 cells are suitable in vitro models for evaluating F3-based radioconjugates. The composition of the cell medium, particularly the presence of Ca^2+^/Mg^2+^, has a marked impact on tracer uptake, most likely because these divalent cations interfere with radioligand binding to cell-surface nucleolin. In addition, factors such as cell density and passage number can influence the observed cellular uptake of F3-based radiotracers, which may be the reason for the variable in vitro results reported across different published studies. Our experiences demonstrate that the preclinical evaluation of F3-radioconjugates in vitro is not straightforward, and that it requires the consideration of multiple parameters that may or may not lead to variable results. Even under optimized conditions, however, the cellular uptake of the investigated F3 radioconjugates remained below 2% of the applied radioactivity, indicating that their performance is currently not sufficient for applications in nuclear medicine. Ongoing work in our group aims to address these limitations in order to enable the implementation of radiolabeled F3 derivatives in (pre)clinical research.

## Figures and Tables

**Table 1:** Half-life (*t*_1/2_) in human serum and in vitro stability of ^111^In-labeled conjugates **DOTA-F3C** and **DOTA-F3N** ^c^.

Compound	Half-life (*t*_1/2_) ^a^ Human Serum	Stability (%) ^b^ In	
		PBS	RPMI 1640
[^111^In]In-**DOTA-F3C**	2.4 (1.4–5.3) h	97.4 ± 0.4	93.3 ± 1.3
[^111^In]In-**DOTA-F3N**	1.5 (1.2–2.0) h	96.1 ± 1.4	93.2 ± 0.8

^a^ mean (95% confidence interval); ^b^ intact radioconjugate after 24 h at 37 °C; data were normalized to 100% at t = 0 h (initial radiochemical purity ≥95%); mean ± standard deviation (SD); ^c^ n = 2 for *t*_1/2_, n = 3 for stability.

## Data Availability

The original contributions presented in this study are included in the article/Supplementary Materials. Further inquiries can be directed to the corresponding author.

## Supplementary Materials

The following supporting information can be downloaded at: https://www.mdpi.com/article/10.3390/ph19040558/s1, Figure S1: Publication timeline of journal articles including the F3 peptide. Figure S2: Structures of the F3 peptide and F3 peptide-based conjugates DOTA-F3C, DOTA-F3N, and FITC-F3N. Scheme S1: Radiolabeling of DOTA-conjugates with ^111^In presented in this work, exemplified by DOTA-F3C. Figure S3: Representative radio-HPLC chromatogram of a co-injection of non-radiolabeled DOTA-F3C with ^111^In-labeled DOTA-F3C. Figure S4: Representative radio-HPLC chromatogram of a co-injection of non-radiolabeled DOTA-F3N with ^111^In-labeled DOTA-F3N. Figure S5: Radio-HPLC chromatograms of [^111^In]In-DOTA-F3C in pooled human serum at 37 °C after 1, 2, and 4 h of incubation. Figure S6: Radio-HPLC chromatograms of [^111^In]In-DOTA-F3N in pooled human serum at 37 °C after 1, 2, and 4 h of incubation. Figure S7: Western blot analysis of a panel of cell lines using an anti-NCL antibody (clone D4C7O). Table S1: Overview of conducted cell assays with MDA-MB-231 cells and ^111^In-radiolabeled F3 peptide-based conjugates. Table S2: Overview of conducted cell assays with H2N cells and ^111^In-radiolabeled F3 peptide-based conjugates. Figure S8: Cell-uptake of [^111^In]In-DOTA-F3C (5 nM) in HUVECs. Figure S9: MDA-MB-231 cells incubated at 37 °C for 120 min in PBS with 0.9 mM Ca^2+^ and without Ca^2+^. Figure S10: Cell-uptake of [^111^In]In-DOTA-F3C (5 nM) in MDA-MB-231 cells seeded in EndoGRO™ medium supplemented with VEGF complete media kit. Figure S11: Cell-associated activity of [^111^In]In-DOTA-F3C (5 nM) in MDA-MB-231 cells, where cells were seeded either in RPMI 1640 + 10% FBS or RPMI 1640 + 10% FBS supplemented with VEGF (20 ng/mL). Figure S12: Cell-uptake assay of [^111^In]In-DOTA-F3C (5 nM) in MDA-MB-231 cells after 60 min, where cells were seeded at different densities. Figure S13: Comparison of consecutive cell assays performed under identical conditions with MDA-MB-231 cells and [^111^In]In-DOTA-F3C (5 nM). References [63,64] cited in Supplementary Materials.

## Abbreviations

AE: Auger electron; Ahx: aminohexanoic acid; DAD: diode array detector; DAPI: 4′,6-diamidino-2-phenylindole; DOTA: 1,4,7,10-tetraazacyclododecane-1,4,7,10-tetraacetic acid; DTPA: diethylenetriaminepentaacetic acid; EC: electron capture; F3: tumor-homing peptide fragment of HMGN2 protein (KDEPQRRSARLSAKPAPPKPEPKPKKAPAKK); FBS: fetal bovine serum; FITC: fluorescein isothiocyanate; HMGN2: high-mobility group nucleosomal binding domain 2; HPLC: high-performance liquid chromatography; HRP: horseradish peroxidase; ID/g: injected dose per gram; LC–MS: liquid chromatography–mass spectrometry; LET: linear energy transfer; MBq: megabecquerel; MS: mass spectrometry; NCL: nucleolin; NOTA: 1,4,7-triazacyclononane-1,4,7-triacetic acid; PBS: phosphate-buffered saline; PenStrep: penicillin–streptomycin; RCP: radiochemical purity; RCY: radiochemical yield; RIPA: radioimmunoprecipitation assay; RPMI: Roswell Park Memorial Institute (medium); SDS-PAGE: sodium dodecyl sulfate–polyacrylamide gel electrophoresis; SPECT: single-photon emission computed tomography; TFA: trifluoroacetic acid; TNBC: triple-negative breast cancer; Tris: tris(hydroxymethyl)aminomethane; TRT: targeted radionuclide therapy; UV/Vis: ultraviolet–visible (spectroscopy);VEGF: vascular endothelial growth factor.
