# Supplementary material for: Revisiting the F3 Peptide: In Vitro Investigations of C- and N-Terminally Modified Peptide Conjugates for Radiotracer Development
Source: Pharmaceuticals (Basel). 2026 Mar 31;19(4):558. doi: 10.3390/ph19040558 (PMC13119255; doi:10.3390/ph19040558)
Supplement: Supplementary file 1 [file pharmaceuticals-19-00558-s001.zip › pharmaceuticals-4203082-supplementary.pdf]

Revisiting the F3 Peptide: In vitro Investigations of C- and N-Terminally Modified Peptide Conjugates for Radiotracer Development

-Supporting Information-

Maximilian Anderla[1,2,3], Marlene Grillmayr[1], Katharina Huemer[1], and Thomas L. Mindt[1,3,4,5]

[1]Institute of Inorganic Chemistry, Faculty of Chemistry, University of Vienna, Josef-Holaubek-Platz 2 and Währinger Straße 42, 1090 Vienna, Austria

[2]Vienna Doctoral School in Chemistry, University of Vienna, Währinger Straße 42, 1090 Vienna, Austria

[3]Ludwig Boltzmann Institute Applied Diagnostics, AKH Wien, Währinger Gürtel 18-20, 1090 Vienna, Austria

[4]Joint Applied Medicinal Radiochemistry Facility, University of Vienna and Medical University of Vienna, 1090 Vienna, Austria

[5]Department of Biomedical Imaging and Image Guided Therapy, Division of Nuclear Medicine, Medical University of Vienna, Währinger Gürtel 18-20, 1090 Vienna, Austria

Keywords: F3 peptide, nucleolin, indium-111, Auger electron therapy, optical/SPECT imaging

Table of Contents

S1 Publication timeline of peer-reviewed journal articles about the F3 peptide....................2

S2 Structures and Schemes....................3

S3 Radio-HPLC chromatograms....................4

S4 Complementary data of stability studies in human serum....................6

S5 Western blot of total NCL levels....................8

S6 Complementary *in vitro* Data....................9

S6.1 Results of cell uptake assays using non-standardized conditions....................9

S6.2 Preliminary experiments with human umbilical vein endothelial cells....................11

S6.3 Effect of Calcium and Magnesium on Cell Attachment....................12

S6.4 Cell culture and assay conditions influence uptake and internalization of F3-based radioconjugate....................12

S6.5 Influence of Cell Density on Cellular Uptake of F3-based Radioconjugate....................13

S6.6 Prolonged cell culture is associated with lower cell uptake....................14

S7 References....................14

S1 Publication timeline of peer-reviewed journal articles about the F3 peptide

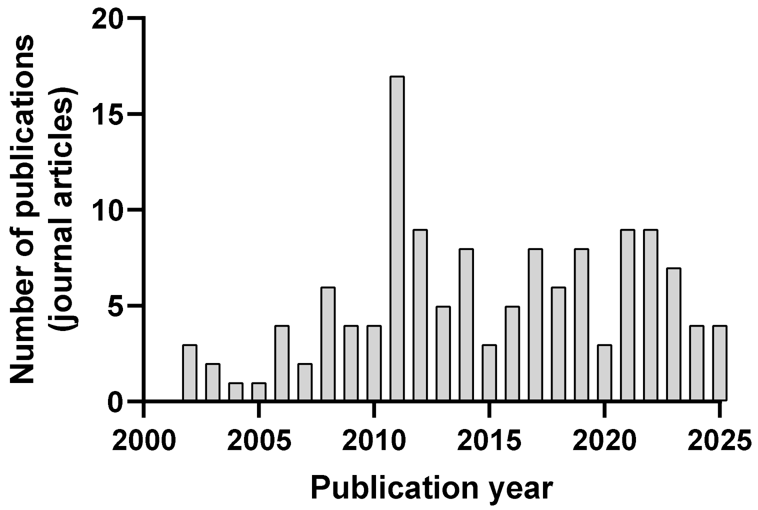

Figure S1. Publication timeline of journal articles including the F3 peptide, generated from a Web of Science™ search using the following keywords: "F3-peptide" OR "F3 peptide" OR “peptide F3”. Relevant articles (138 in total) were selected from an initial dataset of 180 records (search performed on December 6, 2025).

S2 Structures and Schemes

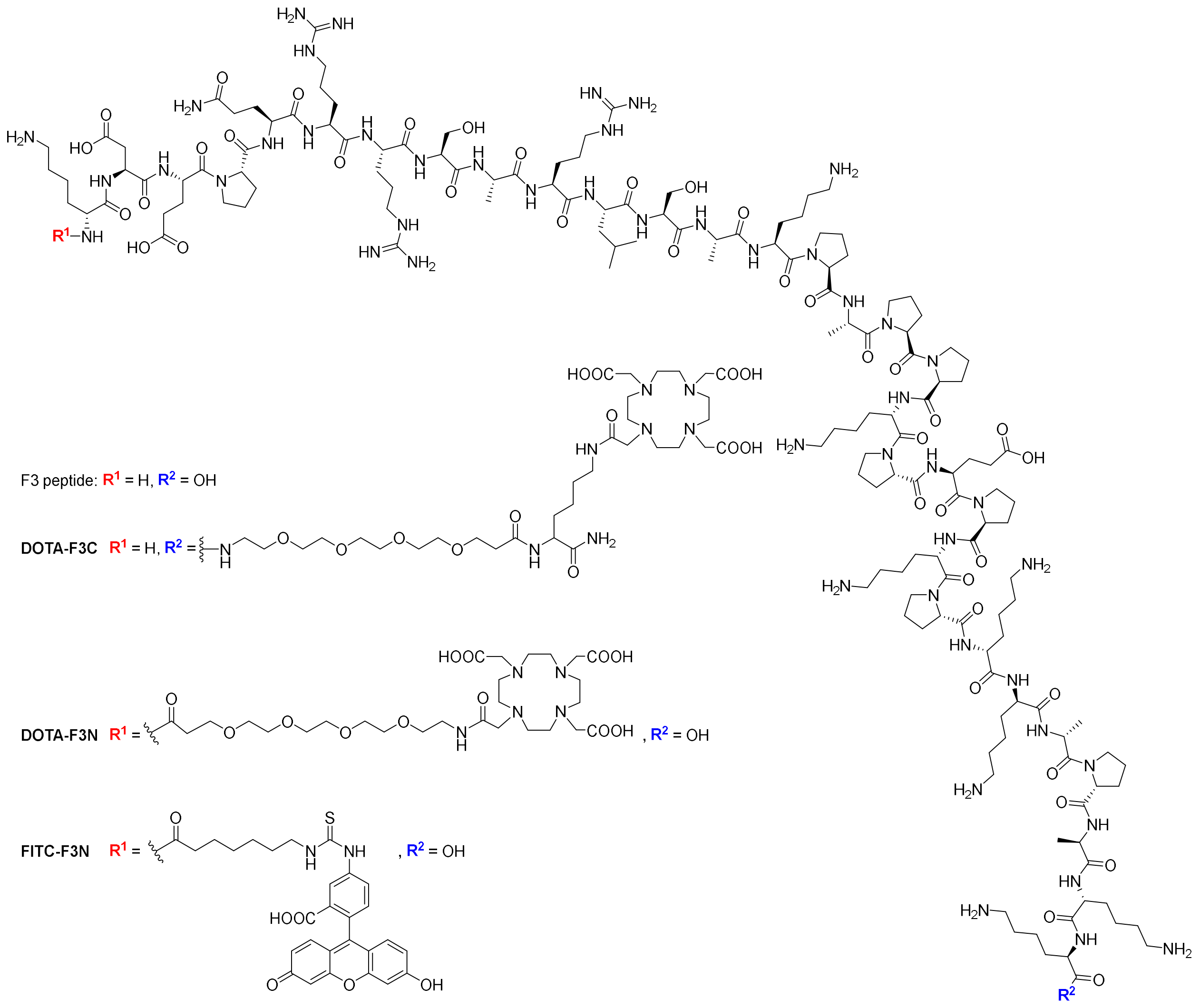

Figure S2. Structures of the F3 peptide and F3 peptide-based conjugates **DOTA-F3C**, **DOTA-F3N**, and **FITC-F3N**.

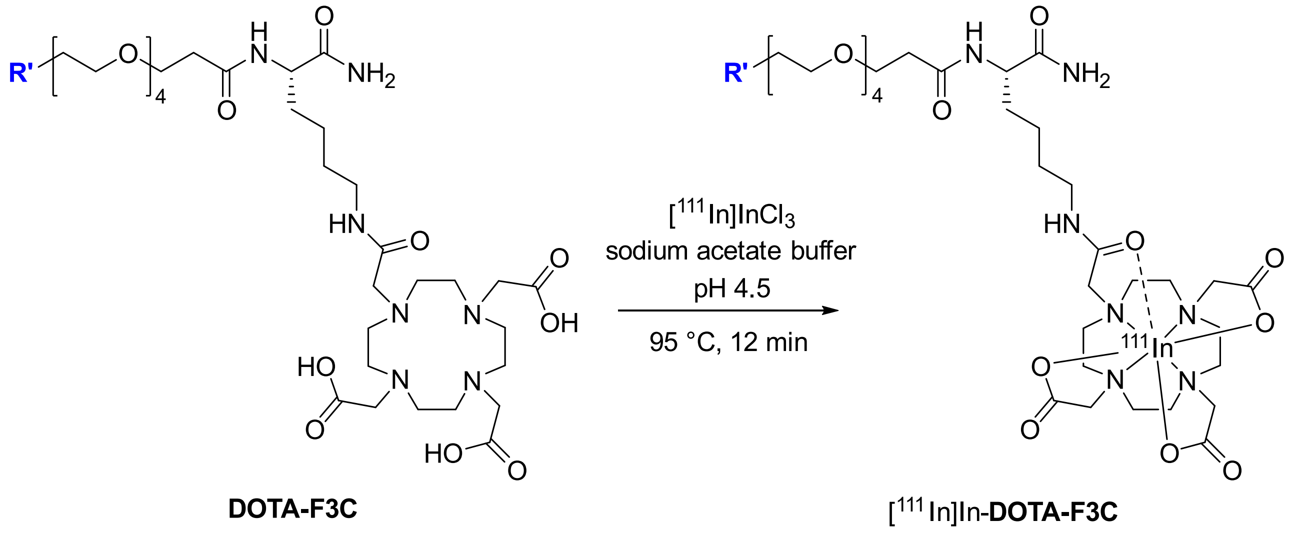

R' = KDEPQRRSARLSAKPAPPKPEPKPKKAPAKK

Scheme S1. Radiolabeling of DOTA-conjugates with ^{111}In presented in this work, exemplified by **DOTA-F3C**.

S3 Radio-HPLC chromatograms

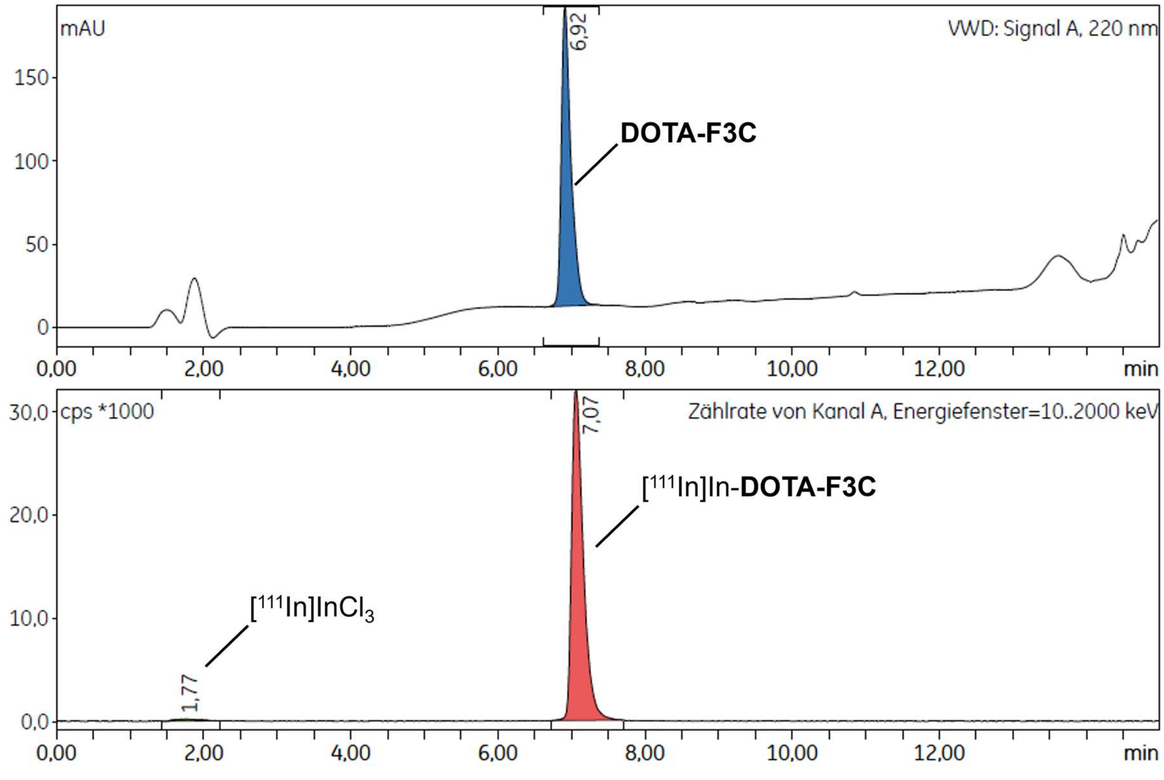

Figure S3. Representative radio-HPLC chromatogram of a co-injection of non-radiolabeled **DOTA-F3C** with ^{111}In-labeled **DOTA-F3C**. UV-channel (top): t_R(**DOTA-F3C**) = 6.92 min; radio-channel (bottom): t_R([^{111}In]InCl$_3$) = 1.77 min, t_R([^{111}In]-**DOTA-F3C**) = 7.07 min, RCY and RCP: 98.7%. The slight shift in retention time between the non-labeled and radiolabeled conjugate is due to the serial alignment of the UV- and radio detector, respectively.

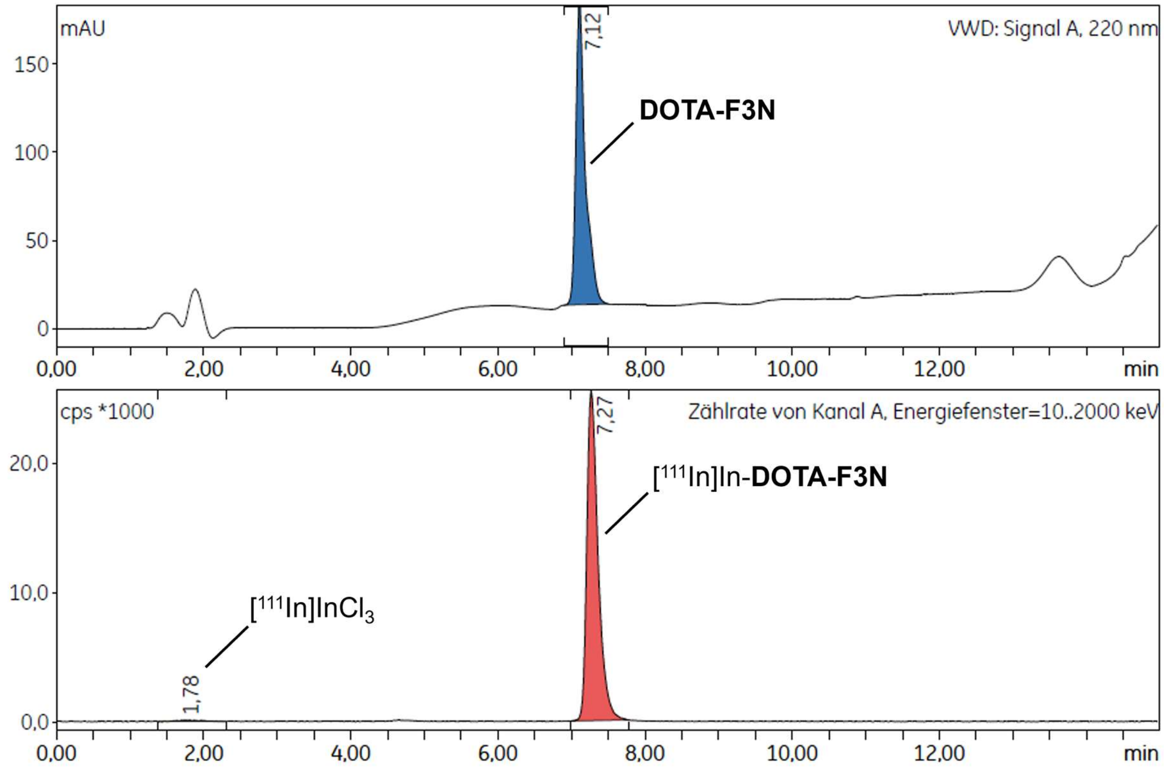

Figure S4. Representative radio-HPLC chromatogram of a co-injection of non-radiolabeled **DOTA-F3N** with ^{111}In-labeled **DOTA-F3N**. UV-channel (top): t_R(**DOTA-F3N**) = 7.12 min; radio-channel (bottom): t_R([^{111}In]InCl$_3$) = 1.78 min, t_R([^{111}In]-**DOTA-F3N**) = 7.27 min, RCY and RCP: 99.3%. The slight shift in retention time between the non-labeled and radiolabeled conjugate is due to the serial alignment of the UV- and radio detector, respectively.

S4 Complementary data of stability studies in human serum

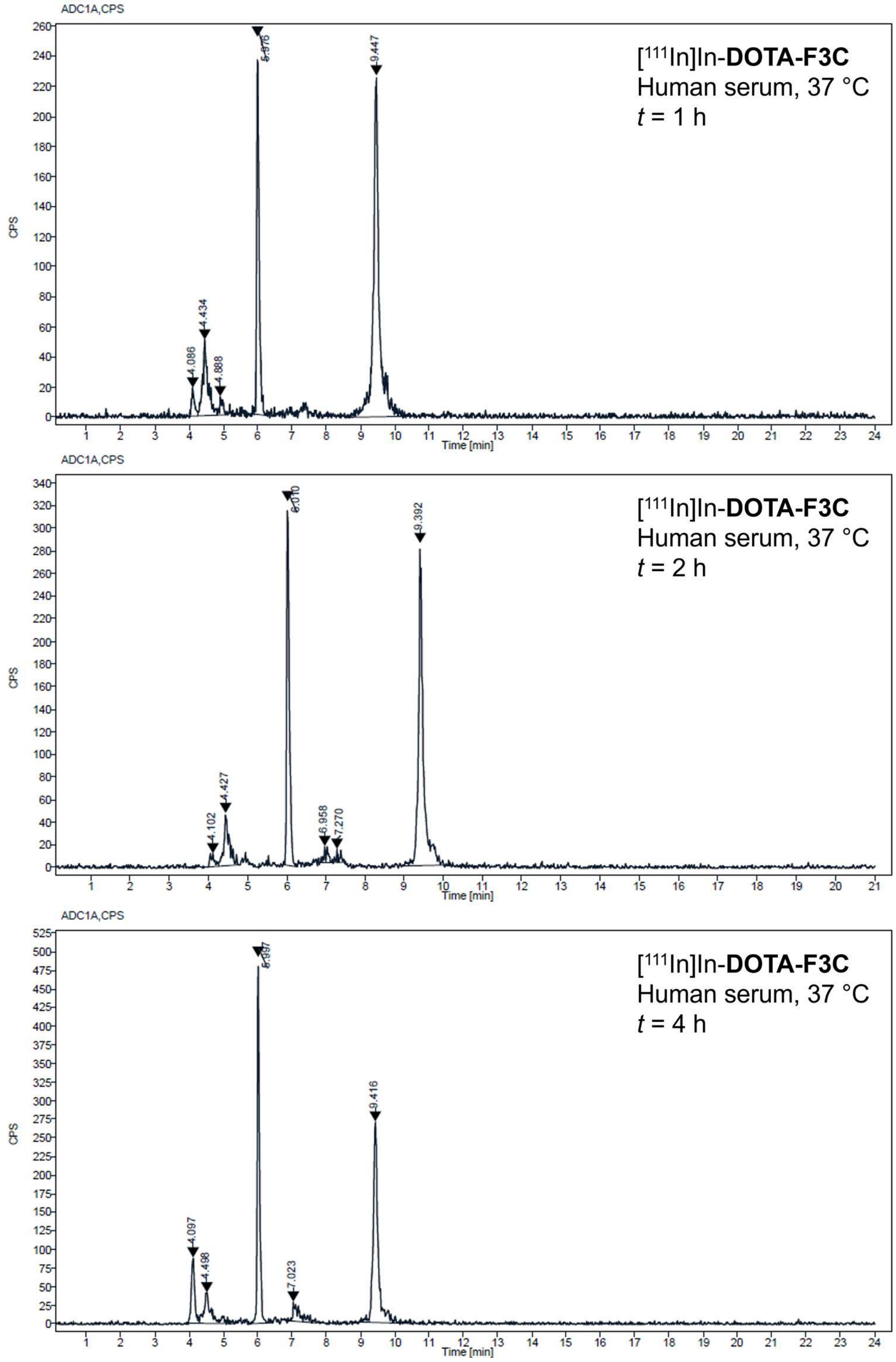

Figure S5. Radio-HPLC chromatograms of [^{111}In]In-**DOTA-F3C** (t_R = 9.4 min) in pooled human serum at 37 °C after 1, 2, and 4 h of incubation. Free, non-labeled ^{111}In and hydrophilic metabolites eluted between 4.1 and 4.5 minutes. Setup: Semipreparative reversed-phase HPLC (gradient: 10–60% acetonitrile + 0.1% TFA over 10 min).

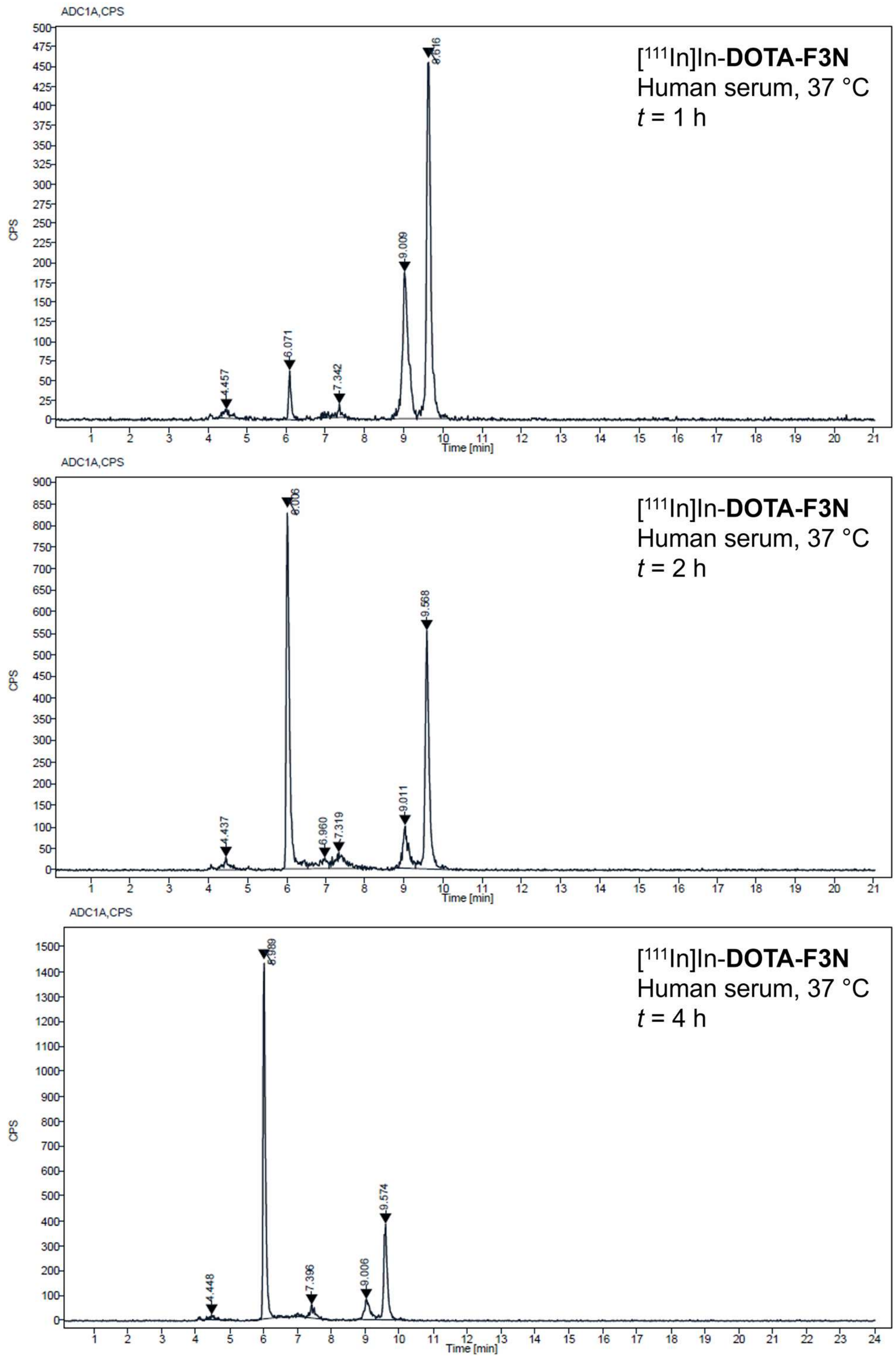

Figure S6. Radio-HPLC chromatograms of [^{111}In]In-**DOTA-F3N** (t_R = 9.6 min) in pooled human serum at 37 ºC after 1, 2, and 4 h of incubation. Free, non-labeled ^{111}In and hydrophilic metabolites eluted between 4.1 and 4.5 minutes. Setup: Semipreparative reversed-phase HPLC (gradient: 10–60% acetonitrile + 0.1% TFA over 10 min).

S5 Western blot of total NCL levels

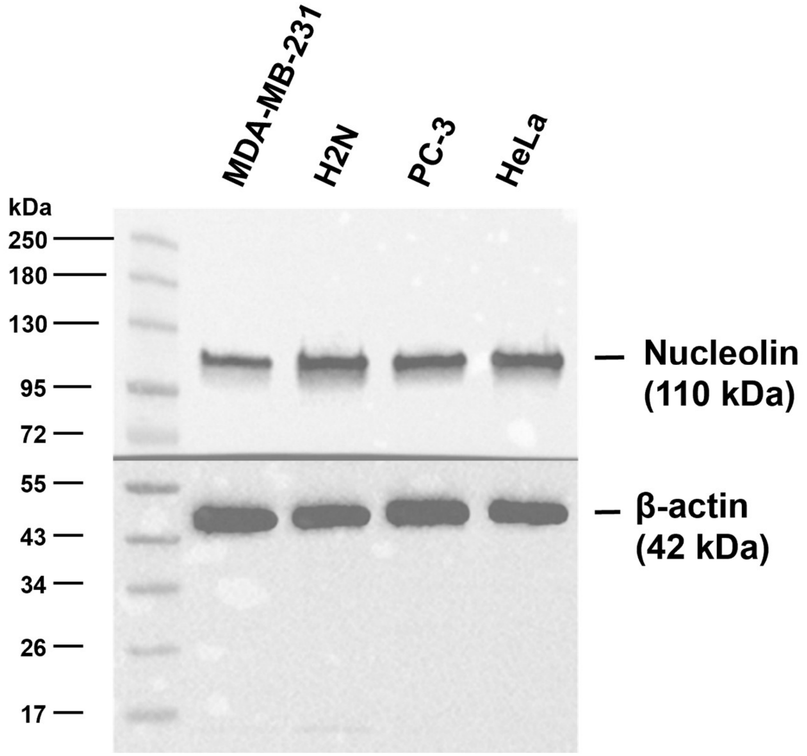

Figure S7. Western blot analysis of a panel of cell lines using an anti-NCL antibody (clone D4C7O). Comparison of total NCL levels using 15 µg protein (whole cell lysate) per lane; antibody dilution 1:2000. To ensure uniform protein input and successful transfer to the membrane, beta-actin was detected as a loading control across all samples.

S6 Complementary *in vitro* Data

S6.1 Results of cell uptake assays using non-standardized conditions

Table S1. Overview of conducted cell assays with MDA-MB-231 cells and ^{111}In-radiolabeled F3 peptide-based conjugates. Assays were performed in triplicates.

Entry	Precursor	Conc./well	Molar activity [MBq/nmol]	Number of cells seeded/well	Blocking[a]	Total bound fraction (%)[b]	Total specific bound fraction (%)[b]
1	**DOTA-F3C**	1 nM	22.4	5×10^5 [c]	1 µM	1.4 ± 0.2*	0.5 ± 0.2*
2	**DOTA-F3N**	1 nM	12.5	3.5×10^5 [c]	-	0.7 ± 0.1**	-
3	**DOTA-F3C**	1 nM	2.5	4×10^4 [d]	5 µM	≤ 0.4	≤ 0.4
4	**DOTA-F3C**	10 nM	2.5	2×10^4 [d]	5 µM	0.9 ± 0.1***	-[e]
5	**DOTA-F3C**	0.1 nM	22	2×10^4 [d]	-	1.1 ± 0.1***	-
6	**DOTA-F3C**	50 nM	22	2×10^4 [d]	-	≤ 0.4	-
7	**DOTA-F3N**	1 nM	16	2×10^4 [d]	-	≤ 0.4	-
8	**DOTA-F3N**	30 nM	7.4	2×10^4 [d]	-	≤ 0.4	-
9	**DOTA-F3C**	0.2 nM	56.6	2×10^4 [d]	1 µM	≤ 0.4	≤ 0.4
10	**DOTA-F3C**	0.5 nM	6.5	5×10^4 [d]	5 µM	0.9 ± 0.1**	-[e]
11	**DOTA-F3C**	5 nM	6.5	5×10^4 [d]	50 µM	1.6 ± 0.2**	0.4 ± 0.2**
12	**DOTA-F3C**	5 nM	47.8	3×10^4 [d]	37 µM[f]	0.5 ± 0.1**	≤ 0.4

[a]F3 peptide (KDEPQRRSARLSAKPAPPKPEPKPKKAPAKK) was employed for blocking; [b]Highest observed value, after *30, **60, ***120, or ****240 minutes; [c]6-well plate; [d] 24-well plate; [e]Non-specific bound fraction was equal or higher than total bound fraction; [f]Preincubation with blocking agent for 30 minutes prior to assay.

Table S2. Overview of conducted cell assays with H2N cells and ^{111}In-radiolabeled F3 peptide-based conjugates. Assays were performed in triplicates.

Entry	Precursor	Conc./well	Molar activity [MBq/nmol]	Number of cells seeded/well	Cells seeded hours prior to assay	Blocking	Total bound fraction (%)[b]	Total specific bound fraction (%)[b]
1	**DOTA-F3C**	0.2 nM	56.6	2 × 10^{4} [c]	24	1 µM	0.4 ± 0.1**	-[e]
2	**DOTA-F3C**	0.5 nM	6.5	5 × 10^{4} [c]	24	5 µM	1.0 ± 0.2**	-[e]
3	**DOTA-F3C**	5 nM	6.5	5 × 10^{4} [c]	24	50 µM	1.6 ± 0.2**	0.7 ± 0.2**
4	**DOTA-F3C**	5 nM	8	5 × 10^{4} [c]	48	20 µM	≤ 0.4	≤ 0.4
5	**DOTA-F3C**	5 nM	14.4	3 × 10^{5} [d]	6	10 µM[f]	1.4 ± 0.4***	0.5 ± 0.2***
6	**DOTA-F3N**	5 nM	13.9	3 × 10^{5} [d]	6	10 µM[f]	≤ 0.4	≤ 0.4
7	**DOTA-F3C**	5 nM	11.1	3 × 10^{5} [d]	18	10 µM[f]	≤ 0.4	≤ 0.4
8	**DOTA-F3N**	5 nM	11.2	3 × 10^{5} [d]	18	10 µM[f]	≤ 0.4	≤ 0.4
9	**DOTA-F3C**	5 nM	4.2	2.5 × 10^{5} [d]	18	10 µM[f]	≤ 0.4	≤ 0.4
10	**DOTA-F3N**	5 nM	4.2	2.5 × 10^{5} [d]	18	10 µM[f]	≤ 0.4	≤ 0.4
11	**DOTA-F3C**	5 nM	1.6	1 × 10^{5} [d]	48	10 µM[f]	1.5 ± 0.2***	1.0 ± 0.2***
12	**DOTA-F3N**	5 nM	1.6	1 × 10^{5} [d]	48	10 µM[f]	1.0 ± 0.1***	≤ 0.4
13	**DOTA-F3C**	10 nM	2.8	1 × 10^{5} [d]	48	20 µM[f]	1.3 ± 0.2***	0.6 ± 0.1***
		25 nM	2.8	1 × 10^{5} [d]	48	20 µM[f]	1.0 ±0.1 ***	≤ 0.4
		50 nM	2.8	1 × 10^{5} [d]	48	20 µM[f]	1.0 ± 0.1***	≤ 0.4
		70 nM	2.8	1 × 10^{5} [d]	48	20 µM[f]	2.0 ± 0.1***	1.2 ± 0.1***
14	**DOTA-F3C**	0.2 nM	14.1	1 × 10^{5} [d]	48	2 µM[f]	1.8 ± 0.2***	1.0 ± 0.2***
15	**DOTA-F3C**	5 nM	18.8	1.5 × 10^{5} [d]	48	-	1.6 ± 0.1***	-

[a]F3 peptide (KDEPQRRSARLSAKPAPPKPEPKPKKAPAKK) was employed for blocking; [b]Highest observed value, after * 30, **60, ***120, or ****240 minutes; [c]6-well plate; [d] 24-well plate; [e]Non-specific bound fraction was equal or higher than total bound fraction; [f]Preincubation with blocking agent for 30 minutes prior to assay.

S6.2 Preliminary experiments with human umbilical vein endothelial cells

The F3 peptide has been shown to primarily accumulate within tumor endothelial cells *in vivo*.[17] A screening assay conducted with human umbilical vein endothelial cells (HUVECs) revealed an intriguing uptake pattern of [^{111}In]In-**DOTA-F3C** (Figure S8). HUVECS were cultured in EndoGRO™-VEGF medium. To exclude potential interference of this medium, it was replaced with PBS two hours prior to the radioligand assay as control experiment. To our surprise, under PBS conditions, a markedly enhanced uptake of the radioligand was observed. After 30 minutes, approximately 13% of the applied radioactivity was associated with the membrane-bound fraction, and around 7% was internalized. In contrast, cell-associated activity remained low (~2%) when the assay was conducted in EndoGRO™-VEGF cell culture medium.

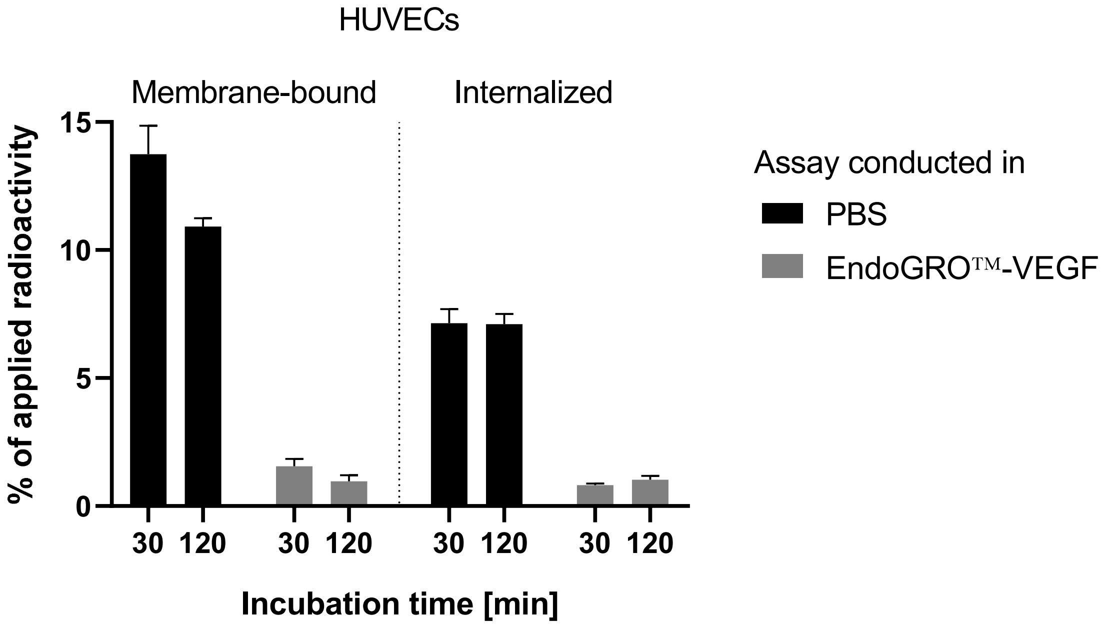

Figure S8. Cell-uptake of [^{111}In]In-**DOTA-F3C** (5 nM) in HUVECs (1.5 × 10^4 cells/well in fibronectin-coated 24-well plates) seeded with EndoGRO™ medium supplemented with VEGF complete media kit. For the PBS condition, the medium was replaced with PBS two hours prior to the addition of the radioconjugate, and incubation was continued at 37 °C. For the EndoGRO™-VEGF condition, the original medium was retained until initiation of the assay. Columns represent mean ± SD (assays were performed in triplicates).

S6.3 Effect of Calcium and Magnesium on Cell Attachment

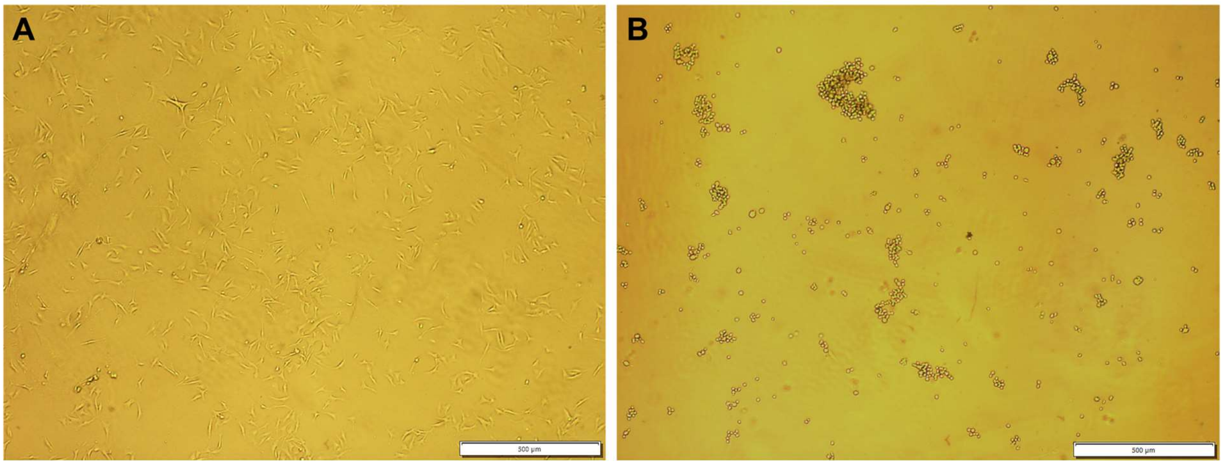

Figure S9. MDA-MB-231 cells incubated at 37 °C for 120 minutes in PBS with 0.9 mM Ca^{2+} (A) and without Ca^{2+} (B). Scale bar: 500 µM.

S6.4 Cell culture and assay conditions influence uptake and internalization of F3-based radioconjugate

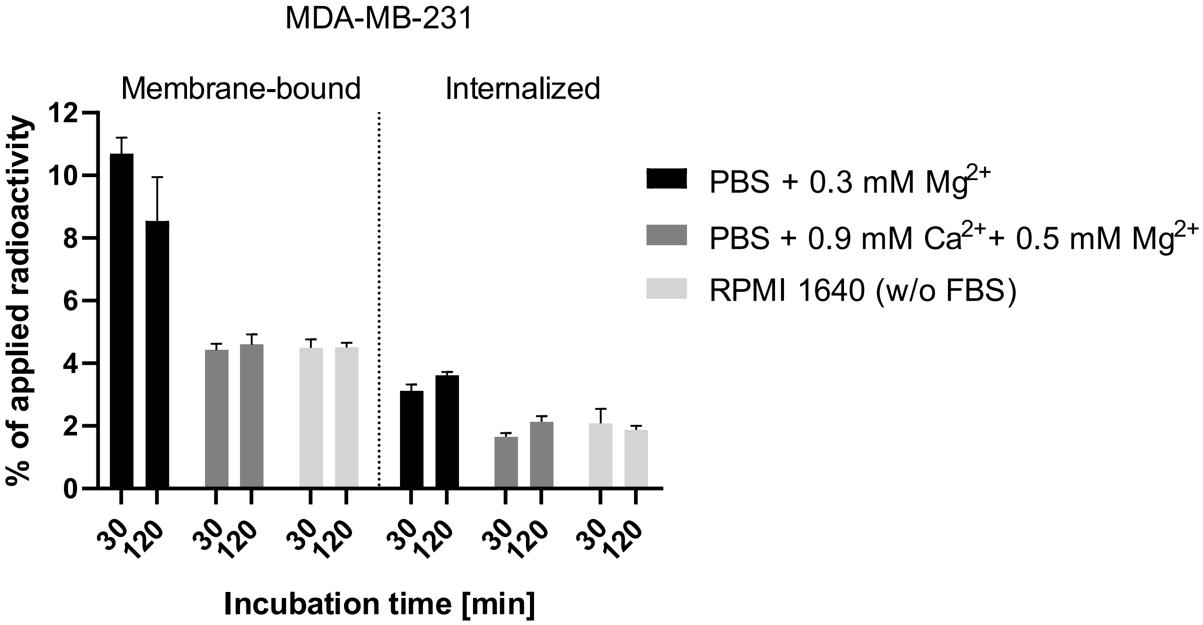

Figure S10. Cell-uptake of [^{111}In]In-**DOTA-F3C** (5 nM) in MDA-MB-231 cells (2 × 10^4 cells/well in 24-well plates) seeded in EndoGRO™ medium supplemented with VEGF complete media kit. Thirty minutes prior to the assay, the medium was replaced either with PBS + 0.3 mM Mg^{2+}, PBS + 0.9 mM Ca^{2+} and Mg^{2+}, or RPMI 1640 (w/o FBS) and incubation continued at 37 °C. Columns represent mean ± SD (assays were performed in triplicates).

Although no specific studies on EndoGRO™-VEGF medium have been reported, VEGF itself has been shown to stimulate the translocation of NCL to the cell surface.[63,64] However, our findings suggest that VEGF alone as supplement is unlikely to account for the increased surface expression of NCL in MDA-MB-231 cells. Control experiments with VEGF addition (20 ng/mL), did not result in elevated [^{111}In]In-**DOTA-F3C** uptake (Figure S11).

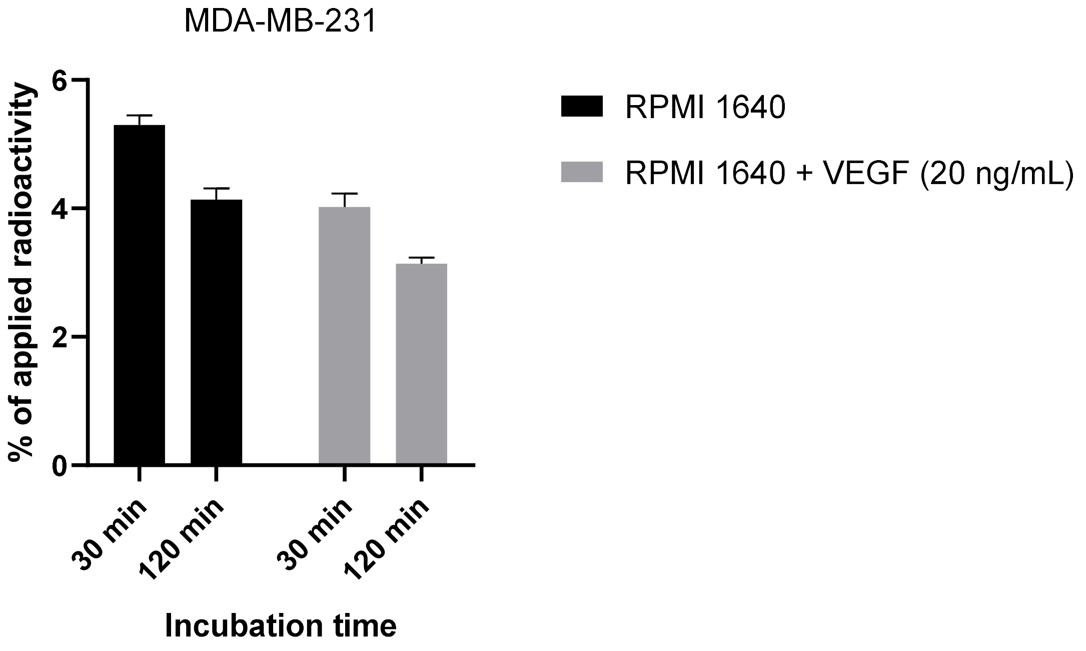

Figure S11. Cell-associated activity of [^{111}In]In-**DOTA-F3C** (5 nM) in MDA-MB-231 cells (2 × 10^4 cells/well, seeded in 24-well plates). Cells were seeded either in RPMI 1640 + 10% FBS or RPMI 1640 + 10% FBS supplemented with VEGF (20 ng/mL). For both conditions, the medium was replaced with PBS + 0.3 mM Mg^{2+} 30 minutes prior to the assay. Columns represent mean ± SD (assays were performed in triplicates).

S6.5 Influence of Cell Density on Cellular Uptake of F3-based Radioconjugate

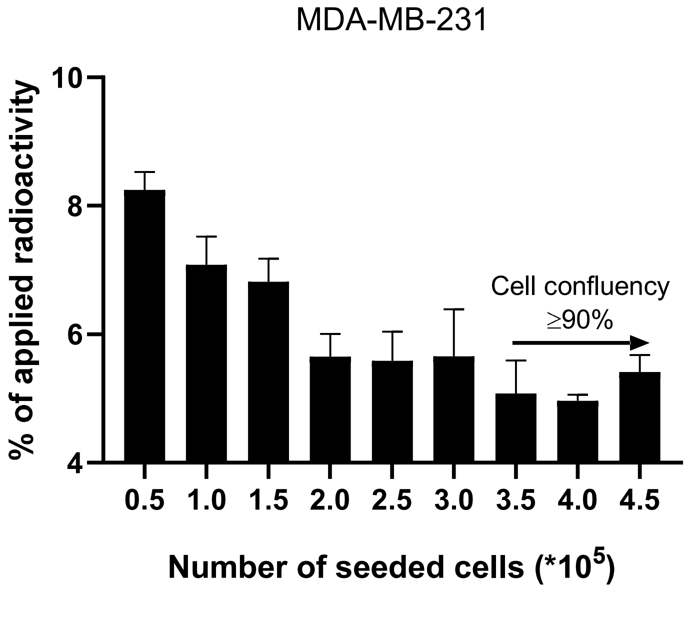

Figure S12. Cell-uptake assay of [^{111}In]In-**DOTA-F3C** (5 nM) in MDA-MB-231 cells after 60 minutes. Cells were seeded 24 hours before the assay at different densities in 6-well plates using EndoGRO™ medium supplemented with VEGF complete media kit. 30 minutes prior to the assay, the medium was replaced with PBS + 0.3 mM Mg^{2+} and incubation continued at 37 °C. Columns represent mean ± SD (*n* = 2 in duplicates).

S6.6 Prolonged cell culture is associated with lower cell uptake

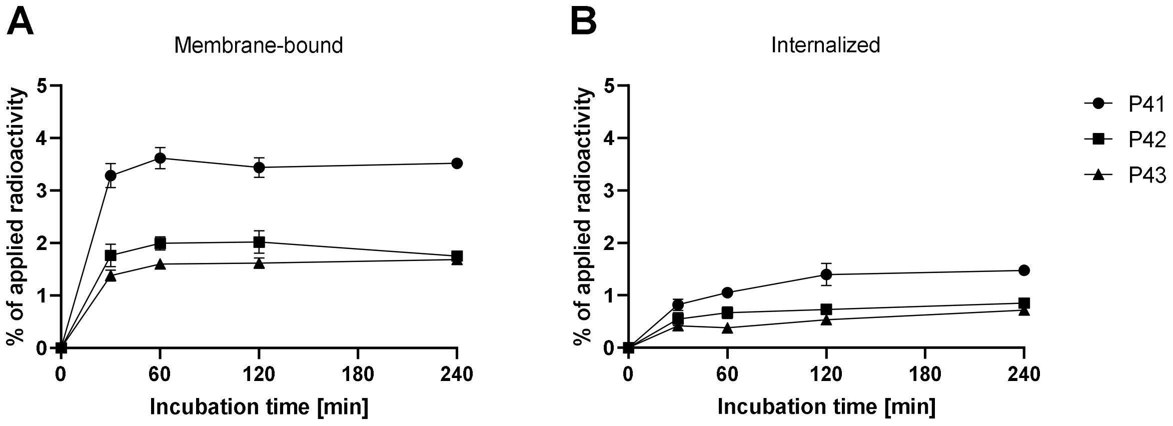

Figure S13. Comparison of consecutive cell assays performed under identical conditions with MDA-MB-231 cells (1 × 10^5 cells/well) and [^{111}In]In-**DOTA-F3C** (5 nM). P stands for passage number. Cells were seeded in EndoGRO™-VEGF and the medium was replaced with RPMI 1640 (w/o FBS) 30 minutes prior to the assay. A) Membrane-bound fraction; B) Total internalized fraction. Data are shown as mean ± SD (assays were performed in triplicates).

S7 References

17. Porkka, K.; Laakkonen, P.; Hoffman, J.A.; Bernasconi, M.; Ruoslahti, E. A Fragment of the HMGN2 Protein Homes to the Nuclei of Tumor Cells and Tumor Endothelial Cells in Vivo. *Proc. Natl. Acad. Sci.* **2002**, *99*, 7444–7449, doi:10.1073/pnas.062189599.
63. Huang, Y.; Shi, H.; Zhou, H.; Song, X.; Yuan, S.; Luo, Y. The Angiogenic Function of Nucleolin Is Mediated by Vascular Endothelial Growth Factor and Nonmuscle Myosin. *Blood* **2006**, *107*, 3564–3571, doi:10.1182/blood-2005-07-2961.
64. Koutsioumpa, M.; Polytarchou, C.; Courty, J.; Zhang, Y.; Kieffer, N.; Mikelis, C.; Skandalis, S.S.; Hellman, U.; Iliopoulos, D.; Papadimitriou, E. Interplay between Avβ3 Integrin and Nucleolin Regulates Human Endothelial and Glioma Cell Migration. *J. Biol. Chem.* **2013**, *288*, 343–354, doi:10.1074/jbc.M112.387076.
